# Sleep fragmentation and heightened inflammation: A latent profile subtype predicting nine-year major depressive disorder severity in the MIDUS study

**DOI:** 10.1016/j.bbih.2026.101332

**Published:** 2026-08-11

**Authors:** Nur Hani Zainal, Sherry A. Beaudreau

**Affiliations:** a National University of Singapore, Department of Psychology, Kent Ridge Campus, Singapore; b Yeo Boon Khim Mind-Science Centre, National University of Singapore, Singapore; c VA Palo Alto Health Care System, Sierra Pacific Mental Illness Research, Education, and Clinical Center, Palo Alto, CA, USA; d Stanford University School of Medicine, Department of Psychiatry and Behavioral Sciences, Stanford, CA, USA; e University of Queensland, School of Psychology, Brisbane, Australia

**Keywords:** Inflammation, Latent profile analysis, Major depression, Precision mental health, Psychoneuroimmunology, Sleep disturbances

## Abstract

**Background:**

Cross-sectional and standard approaches to examining proinflammatory activity and sleep disturbances separately hinder understanding of their additive, long-term impacts on major depressive disorder (MDD). The current study used latent profile analysis (LPA) to identify multimodal neuroimmune profiles and to investigate whether subtypes predicted 9-year MDD severity in a community-dwelling adult sample.

**Method:**

Community adults (*N* = 306) completed psychiatric interviews to measure MDD symptoms at baseline and 9-year follow-up. The Pittsburgh Sleep Quality Index and eight-day actigraphy captured subjective and objective sleep disturbances, respectively. Nine log-transformed inflammatory markers–including C-reactive protein, E-selectin, intercellular adhesion molecule-1, fibrinogen, interleukin-6, interleukin-8, interleukin-10, and tumor necrosis factor-α–were assayed. An LPA with an interpretable 13-variable core set was conducted, with model selection based on various metrics, including split-sample Adjusted Rand Index (ARI) stability.

**Results:**

The two-profile solution provided the best fit (ARI = 0.948). Profile 1 (*n* = 212) was marked by adequate sleep and low inflammation; Profile 2 (*n* = 94) by insufficient sleep and high inflammation. Profile 2 membership was a strong predictor of greater 9-year MDD severity (Cohen's *d* = 0.244, *p* = .039) after controlling for baseline demographics and MDD severity. This effect remained practically significant in the completely adjusted model (*d* = 0.208). LPA had superior performance than variable-centered approaches on parsimony-based model fit.

**Conclusions:**

A neuroimmune profile characterized by proinflammatory activity and insufficient, fragmented sleep functioned as a distal risk factor of MDD symptoms. A person-centered, multimodal risk stratification approach may enhance targeted prevention of long-term MDD.

## Highlights

• LPA found two neuroimmune profiles linking fragmented sleep and inflammation.
• Profile 2 (high inflammation, fragmented sleep) predicted higher 9-year MDD.
• This predictive pattern remained significant over and above baseline MDD severity.
• LPA performed better than regression, K-means, LASSO, and composite models.
• Split-sample replication yielded high stability (Adjusted Rand Index = 0.948).

## 1 Introduction

Major depressive disorder (MDD) is a common mental health disorder with an estimated lifetime prevalence of 2–13% globally (Abdoli et al., 2022; Zhao et al., 2021). As a leading cause of disability worldwide (Chen et al., 2025; Friedrich, 2017; Meshkat et al., 2025), greater MDD severity has been entwined with comorbid mental and physical illnesses, cognitive dysfunction, interpersonal problems, and work impairments (Johnston et al., 2019; Penedo et al., 2025; Snyder, 2013). Societally, the economic costs of medication-treated MDD could exceed $90 billion in the U.S. alone, including increased healthcare expenditures (Zhdanava et al., 2021). These well-established correlates of MDD symptoms highlight the importance of identifying long-term risk factors of MDD symptoms early to inform evidence-based and targeted prevention and intervention efforts.

Sleep disturbances might be an integral long-term risk factor for MDD symptoms. Chronic daytime dysfunction and insomnia symptoms, such as longer sleep onset latency (SOL), shorter total sleep time (TST), and greater wake after sleep onset (WASO), could perpetuate emotion dysregulation, hyperarousal, repetitive negative thinking, and social dysfunction (Dressle and Riemann, 2023; Harvey, 2011; Spielman et al., 1987). These psychological and social processes might coincide with biological mechanisms underlying circadian sleep-wake rhythms, which regulate neurochemicals (e.g., melatonin, serotonin) critical for rapid eye movement (REM) and non-REM sleep (Riemann et al., 2023; Won et al., 2021). Across long durations, these biopsychosocial pathways would likely culminate in more MDD symptoms.

Various meta-analyses have buttressed the assertion that sleep disturbances precede and predict greater MDD severity. Nondepressed adults with (vs. without) daytime dysfunction and insomnia symptoms (e.g., long and short TST) were twice to thrice as likely to develop clinical depression later (Baglioni et al., 2011; Hertenstein et al., 2019; Li et al., 2016; Zhai et al., 2015). Moreover, similar prospective patterns generalized to youth populations (Marino et al., 2021). However, most research on this topic has been cross-sectional, limiting causal inference (Savitz and Wellenius, 2023). In addition, overreliance on self-reports, such as the Pittsburgh Sleep Quality Index (PSQI; Buysse et al., 1989), might lead to discrepancies compared with actigraphy-based wearable sensors (Lubas et al., 2022). Finally, the examination of sleep disturbances as distal risk factors of MDD symptoms would remain incomplete without integrating them with key biomarkers.

Proinflammatory activity could be a viable distal risk factor of MDD symptoms that operate in tandem with sleep disturbances and serve as long-term predictors. Cytokines (e.g., interleukin-6 [IL-6] and tumor necrosis factor-alpha [TNF-α]) might induce greater expression of indoleamine 2,3-dioxygenase (IDO), thereby reducing tryptophan availability for serotonin production and producing more toxic kynurenine metabolites (Haroon et al., 2020; Hunt et al., 2020). Such proinflammatory activity would likely interact with chronic stressors across environments, leading to long-term difficulties with focus, motivation, and social functioning (Slavich, 2020; Slavich and Irwin, 2014). Across time, these mechanisms might adversely affect endocrine and neural pathways, such as overactivation of the hypothalamic–pituitary–adrenal (HPA) axis (Miller et al., 2009; Miller and Raison, 2016). Together, higher proinflammatory activity might predict more severe future MDD symptoms.

Consistent with these theories, several meta-analyses of cross-sectional and longitudinal studies have shown that greater proinflammatory activity was associated with greater MDD severity. Compared with healthy controls, depressed individuals showed higher levels of C-reactive protein (CRP), IL-6, IL-10, intercellular adhesion molecule-1 (ICAM-1), and TNF-α (Dowlati et al., 2010; Haapakoski et al., 2015; Kohler et al., 2017; Osimo et al., 2020; van Agtmaal et al., 2017). Research has also found that higher plasma fibrinogen levels, indicative of vascular diseases, have been correlated with more depressive symptoms (Hattori et al., 2015; Martins-de-Souza et al., 2014). Two meta-analyses of longitudinal studies have established that higher CRP and IL-6 levels precede and predict greater depression severity (Mac Giollabhui et al., 2021; Valkanova et al., 2013). The present study built on this literature by examining the role of two other biomarkers in predicting future MDD symptoms. Interleukin-8 (IL-8), an important chemokine, was studied given its potential implications and its understudied role in the pathogenesis of depression (Lisowska, 2025). Relatedly, E-selectin, which has been shown to reflect endothelial and microvascular dysfunction, might be a key etiological factor in MDD symptoms (Waclawovsky et al., 2025). Together, higher levels of CRP, E-selectin, IL-6, IL-8, ICAM-1, and TNF-α, coupled with compensatory elevations in the anti-inflammatory cytokine IL-10, might precede an increase in MDD severity.

Accordingly, integrating proinflammatory biomarkers, objective and subjective sleep indices, and relevant clinical variables within the same study would necessarily increase the data set's dimensionality when studying the etiology of MDD symptoms. Traditional ordinary least squares regression is ill-suited to manage high-dimensional data sets because it can lead to a singular (i.e., non-invertible) matrix, resulting in unstable estimates (Shakeel et al., 2023). High intercorrelations among the variables (multicollinearity; Difrancesco et al., 2022) could also contribute to such problems. Latent profile analysis (LPA) addresses these issues by adopting a person-centered rather than a variable-centered perspective, enabling the detection of unobserved (or latent) subtypes within a population (Lanza et al., 2013; Spurk et al., 2020; Van Lissa et al., 2023). By detecting well-defined latent profiles based on key inflammation, sleep, and clinical variables, risk stratification could be conducted to determine the prognostic value of profile membership in predicting long-term MDD symptoms. Together, elucidating subtypes that might be vulnerable to a prolonged MDD illness course via LPA could advance precision mental health (Zainal and Newman, 2021) and inform targeted prevention strategies.

Therefore, we tested these aims in a community-dwelling adult sample from mid- and late-life recruited in the Midlife Development in the United States (MIDUS) study. Based on the theories, research, and logic discussed, the present study aimed to use LPA to identify profiles of proinflammatory activity, sleep disturbances, and relevant clinical variables that function as distal risk factors for MDD symptoms. First, we hypothesized that specific latent profiles would be observed using a high-dimensional set of variables that included objective and subjective sleep, inflammation, and related clinical variables. Second, we expected that profile membership would significantly predict MDD severity nine years later, even after controlling for baseline MDD severity in the latent profile and key demographic covariates.

## 2 Method

### 2.1 Procedures

In the MIDUS Biomarker project (Dienberg Love et al., 2010), some participants underwent an overnight visit at a regional Clinical Research Unit at one of the participating MIDUS universities, during which standardized actigraphy, biomarker, and self-report measures were administered (Friedman et al., 2009; Hansen et al., 2025). Using harmonized procedures, the MIDUS biomarker protocol was conducted at baseline for participants invited to attend a two-day on-site data collection session to measure proinflammatory biomarkers and sleep disturbances using both actigraphy and self-reports (Dienberg Love et al., 2010). The MIDUS project also included the administration of clinical interviews using the Composite International Diagnostic Interview-Short Form (CIDI-SF; Kessler et al., 1998). Other self-reports were also administered to assess various health variables essential to consider when identifying latent profile membership that might predict nine-year MDD severity.

### 2.2 Measures

**Proinflammatory biomarkers.** Eight inflammatory biomarkers were measured: IL-6; IL-8; IL-10; TNF-α; fibrinogen; CRP; E-selectin; and ICAM-1. Following standard procedures, these biomarkers were obtained from post-fasting venous blood samples, centrifuged, and stored at −60 to −80 degrees Celsius prior to transport on dry ice to the designated laboratories for uniform batch processing (Dienberg Love et al., 2010). IL-6 was assayed using two methods: (i) high-sensitivity enzyme-linked immunosorbent assay (ELISA; Quantikine®, R&D Systems; University of Wisconsin, 2024; Zainal, 2025), which assessed the absorbance of serum IL-6 at 490 nm; (ii) Meso Scale Discovery (MSD) V-Plex Cytokine Panel electrochemiluminescence immunoassay (Aw and Zainal, 2025), which used 96-well plates for storage in conjunction with a sector imager that estimated IL-6, IL-8, IL-10, and TNF-α levels. For other biomarkers, plasma samples were shifted to anticoagulant-filled laboratory tubes and centrifuged prior to assay on a BNII nephelometer (University of Wisconsin, 2024; Zainal and Newman, 2021). The nephelometer measured CRP (particle-enhanced immunonephelometric assay) and fibrinogen (N Antiserum to Human Fibrinogen) by forming antibody-antigen immune complexes that shift light scatter, thereby estimating serum levels precisely. E-selectin and ICAM-1 were quantified by enzyme-linked immunosorbent assay. These procedures were standardized, and measurements were duplicated to retain precision and reproducibility (Dienberg Love et al., 2010).

**Sleep disturbances.** Actigraphy sleep phase markers were collected during an eight-day protocol using a Mini Mitter Actiwatch-64 wrist-worn device that tracked activity counts (physical activity), wake bouts (nighttime awakenings), and sleep bouts (nap frequency). After the two-day biomarker on-site data collection protocol, participants wore the actigraphy from Tuesday morning until the following Tuesday morning. Sleep diaries were part of the actigraphy procedure to record the start of sleep, while the actigraphy device captured other metrics. An accelerometer was built into the actigraph, which measured activity counts across 30-s to 1-min epochs, and established sleep-wake algorithms (e.g., Cole-Kripke) were harnessed to categorize periods as sleep or wake phases. Key sleep phase metrics were captured: wake time (counts and %), wake bouts, WASO, TST, SOL, sleep efficiency, sleep bouts, and activity counts. TST was the epoch-derived sleep period from sleep onset to final awakening. WASO was the entire epoch-derived wake time between sleep onset and final awakening. SOL was the epoch-based period between the diary-recorded bedtime and the first episode of prolonged sleep. Sleep efficiency was defined as the ratio of TST to time in bed. Values of all actigraphic metrics were averaged across the seven days for each participant to assess trait-level sleep patterns. Variability metrics were not computed to maintain interpretability and parsimony.

The Pittsburgh Sleep Quality Index (PSQI) was used in the biomarker protocol to measure self-reported sleep disturbances in the past month (Buysse et al., 1989). Seven components of sleep were assessed, each captured by multiple items: subjective sleep quality; sleep latency; sleep duration; habitual sleep efficiency; sleep disturbances; use of sleep medication; and daytime dysfunction. Higher scores denoted greater insomnia severity. The PSQI showed good internal consistency (McDonald's ω = .710 in the present study), high retest reliability, and strong construct validity (Buysse et al., 2008; Dietch et al., 2016). Normative data for the PSQI have also been well established across diverse samples (Beaudreau et al., 2012; Spira et al., 2012).

**Clinical interviews.** The CIDI-SF clinical interviews, aligned with the Diagnostic and Statistical Manual-Third Edition-Revised (DSM-III-R; American Psychiatric Association, 1987), yielded dimensional scores for MDD (range: 0–7; ω = .93 at baseline and ω = .90 at nine-year follow-up), Generalized Anxiety Disorder (GAD) (range: 10–40; ω = .97), and Panic Disorder (PD) (range: 0–6; ω = .92). Higher scores on the CIDI-SF indicated greater symptom severity. At baseline and nine-year follow-up, the MDD scale assessed anhedonia, appetite issues, concentration difficulties, depressed mood, feelings of worthlessness, and suicide ideation for at least two weeks in the past 12 months. Items that captured sleep problems were excluded from the primary analyses to avoid redundancy with sleep variables included in the LPA used to characterize participants. The GAD scale measured worry-related symptoms, such as irritability, muscle tension, and restlessness. The PD scale captured cued and non-cued panic symptoms, such as chest tightness, derealization, and hot flashes. These dimensional scales have shown good reliability and strong construct validity (Gigantesco and Morosini, 2008; Ng et al., 2024). The CIDI-SF also has acceptable reliability and validity for diagnosing conditions, which can be summed to yield psychiatric diagnostic counts (Zainal and Newman, 2019).

**Self-reports.** Substance use disorder (SUD) severity was measured with a MIDUS-specific scale using eight items that captured SUD symptoms (range: 28–89; ω = .95), such as cravings, excessive use, and negative consequences on relationships or work (Miller et al., 2023). In addition, participants reported the counts of drugs used for menopause (range: 0–6), menstrual symptoms (range: 0–3), and aging-related worries (range: 3–12). The number of chronic cardiovascular, dental, endocrine, gastrointestinal, integumentary, metabolic, and respiratory illnesses they had experienced in the past 12 months (range: 0–30) was assessed. Counts of medication use (range: 0–24) and supplement consumption (range: 0–23) in the past 30 days were measured.

### 2.3 Data analyses

**Fig. 1:**
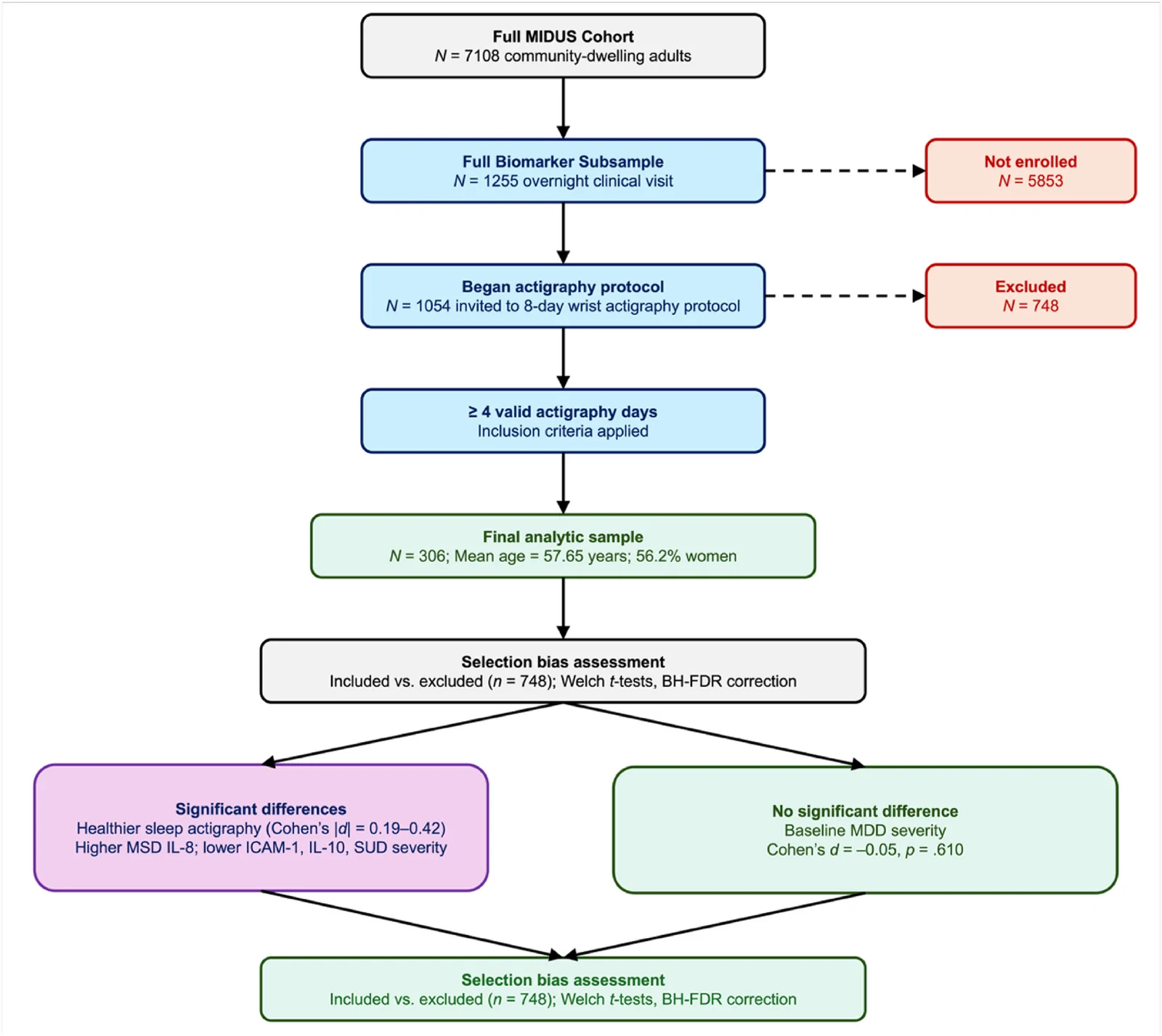
Participant flow and selection bias assessment. *Note*. MIDUS = Midlife Development in the United States; MDD = major depressive disorder; BH-FDR = Benjamini-Hochberg false discovery rate; ICAM-1 = intercellular adhesion molecule-1; IL = interleukin; MSD = Meso Scale Discovery; SUD = substance use disorder. The full MIDUS cohort had *N* = 7108. The Biomarker subsample (*N* = 1255) completed an overnight clinical research visit. Participants invited to the actigraphy protocol (*N* = 1054) wore a Mini Mitter Actiwatch-64 wrist device for eight days; those with fewer than four valid days were excluded, yielding the final analytic sample (*N* = 306). The excluded group (*n* = 748) was compared against the included subsample on all 42 study variables using Welch's independent-samples *t*-tests with Benjamini-Hochberg false discovery rate correction.

Fig. 1 provides a schematic flowchart of how the present study arrived at the final analytic sample of *N* = 306. We natural log-transformed all nine biomarkers (CRP, ELISA IL-6, E-Selectin, Fibrinogen, ICAM-1, MSD IL-6, MSD IL-8, MSD IL-10, and TNF-α) before analysis due to positive skewness, following research recommendations (West, 2022). Furthermore, CRP samples that fell below the BNII Nephlometer identification limit were re-assayed using the Meso Scale Discovery high-sensitivity kit. Table S1 in the online supplemental materials (OSM) lists assay equipment, catalog numbers, manufacturers, and reagent kit numbers. We used Shapiro-Wilk tests (Table S2) to evaluate the distributional characteristics of the four key actigraphy-based and self-reported sleep markers included in the LPA. As typically seen (Wallace et al., 2018), all four sleep markers showed significant non-normal distributions.

Missing data were handled using random forest imputation, which accommodates non-linear relationships and mixed data types (Tang and Ishwaran, 2017), as in the present dataset. The analytic sample (*n* = 306) included those participants with at least four days of complete actigraphy data. To assess selection bias, the excluded sample (*n* = 748) was contrasted against the included subsample on all 42 original study variables using the Welch's independent-samples *t*-tests with the Benjamini-Hochberg false discovery rate (FDR) adjustment (Table S3). To evaluate multicollinearity, associations among the inflammatory markers were regarded as low enough to rule out collinearity concerns (Table S4).

Latent profile analysis (LPA) was performed in *R* using the *tidyLPA* package (Rosenberg et al., 2018), which executes Gaussian mixture models through the *mclust* engine (Oberski, 2016). Because the indicators were continuous, LPA was chosen instead of latent class analysis. To explore optimal model fit, three variance-covariance structures (EEI, VII, and EII) were estimated across 2- to 6-profile solutions (Table S5), ensuring a minimum recommended cases-to-parameters ratio of 10:1 was maintained throughout the analysis (Nylund-Gibson and Choi, 2018). Model selection relied on the Akaike/Bayesian Information Criterion (AIC/BIC), average posterior classification probability, and entropy. Based on these criteria, the VII (variable volume with spherical covariance) model was chosen as the optimal structure. The 2-profile solution was subsequently selected due to alignment of fit indices with replication stability, which was evaluated using the split-sample Adjusted Rand Index (ARI; Steinley, 2004) analysis (Table S6). Specifically, the 2-profile VII solution generated an average ARI of .948, reflecting excellent cross-sample replicability.

To resolve concerns about model stability, given the ratio of indicators to sample size, we performed a sensitivity analysis contrasting the original 42-variable model to the retained 13-variable core set across all profile solutions (Table S7). These two specifications produced similar average posterior probabilities, balances, entropies, and parsimony values. However, the 13-variable core model showed similar or superior ARI outcome across most solutions. To assess the incremental value of the LPA approach, benchmark contrasts (Lu and Petkova, 2014; Nylund-Gibson and Choi, 2018) were conducted against variable-centered multiple regression, three composite-score models, K-means clustering, and least absolute shrinkage and selection operator (LASSO) regression (Table S8). Another sensitivity analysis assessed whether additive and multiplicative interaction terms between sleep efficiency and inflammation yield superior performance in predicting 9-year MDD severity using LPA profile membership (Table S9). Clinically relevant benchmarks used to interpret the meaning of “elevated” or “high” inflammation, and “fragmented” or “poor” sleep are recorded in Table S10 and were derived empirically. These thresholds were purely descriptive and were not included in the LPA framework's categorization process. Finally, the 2-profile solution was entered as a predictor of 9-year MDD severity in a multiple linear regression model, controlling for age, baseline MDD severity, education, race/ethnicity, sex, number of chronic conditions, medications, psychiatric diagnoses, and vitamin supplements (Malhi and Mann, 2018). Baseline MDD severity was retained as an LPA indicator because it is theoretically integral to characterizing the neuroimmune subtype–not merely a covariate–consistent with established precision psychiatry frameworks (Nylund-Gibson and Choi, 2018); its subsequent inclusion as a covariate in the predictive model follows three-step mixture modeling guidelines specifically designed to avoid construct conflation between profile formation and outcome prediction (Asparouhov and Muthén, 2014).

## 3 Results

### 3.1 Participants

Community-dwelling adults enrolled in the longitudinal MIDUS project (Ryff et al., 2017). The MIDUS study is a nationwide prospective cohort study that began in 1995 and included over 7000 noninstitutionalized middle-aged adults in the U.S. to investigate how biopsychosocial variables are linked to diverse age-associated health outcomes across adulthood (Apsley et al., 2024; Brim et al., 2020; Friedman et al., 2009). The present study initially focused on the subset of the initial 1255 participants enrolled in the MIDUS Biomarker protocol (Dienberg Love et al., 2010) and further concentrated on those who began the actigraphy protocol, as this subsample (*N* = 306) aligned with the research aims. Participants in the final analytic sample (*N* = 306) were 57.65 years on average (*SD* = 11.83), with 56.2% women and 43.8% men (Table 2). Most participants were White (94.1%), and the remaining 5.9% either did not disclose their race or indicated they were multiracial, African American, Native American, or “other” race. About two-fifths (41.8%) of participants were college-educated, whereas the remaining 58.2% had some college, a high school diploma, or did not complete high school (see Table 2).

**Table 1:** Between-profile differences on the 13 core latent profile analysis indicators: Profile 1 (low sleep fragmentation, low inflammation) versus profile 2 (high sleep fragmentation, high inflammation) (*N*= 306).

Group	Profile 1 (*n* = 212)		Profile 2 (*n* = 94)		Effect Size	
Variable	*M*	*(SD)*	*M*	*(SD)*	Cohen's *d*	*p*
Sleep efficiency (SE; min)	85.22	(4.99)	75.38	(10.88)	1.35	< .001
Wake after sleep onset (WASO; min)	39.49	(15.63)	54.98	(23.58)	−0.84	< .001
Sleep onset latency (SOL; min)	18.04	(12.65)	40.95	(29.60)	−1.18	< .001
PSQI Global Score	5.24	(2.77)	7.29	(4.20)	−0.63	< .001
ELISA CRP Levels	0.84	(0.51)	1.52	(0.87)	−1.06	< .001
ELISA IL-6 Levels	1.05	(0.35)	1.56	(0.53)	−1.24	< .001
MSD TNF-α Levels	1.10	(0.17)	1.29	(0.25)	−0.93	< .001
ELISA E-Selectin Levels	3.65	(0.44)	3.81	(0.60)	−0.32	.024
ELISA Fibrinogen levels	5.76	(0.22)	5.87	(0.28)	−0.45	.001
ELISA ICAM-1 Levels	5.49	(0.28)	5.67	(0.36)	−0.58	< .001
MSD IL-6 Levels	0.56	(0.19)	0.97	(0.51)	−1.25	< .001
MSD IL-8 Levels	2.66	(0.29)	2.88	(0.46)	−0.65	< .001
MSD IL-10 Levels	0.21	(0.09)	0.33	(0.29)	−0.67	< .001

*Note*. CRP = C-reactive protein; ELISA = enzyme-linked immunosorbent assay; ICAM-1 = Intercellular Adhesion Molecule-1; IL = interleukin; min = minutes; MSD = Meso Scale Diagnostics; PSQI = Pittsburgh Sleep Quality Index; SE = sleep efficiency; SOL = sleep onset latency; TNF-α = tumor necrosis factor-alpha; WASO = wake after sleep onset. *M* = mean; *SD* = standard deviation; *d* = Cohen's *d*; *p* = probability value. Profile 1 (*n* = 212) and Profile 2 (*n* = 94) were derived from the 2-profile solution of the latent profile analysis using 13 core variables. All biomarker variables are presented in log-transformed units consistent with the analytic approach. Between-profile differences were evaluated using Welch's independent-samples *t*-tests, which do not assume equality of variance and are appropriate given the unequal profile sizes. Effect sizes are reported as Cohen's *d*, computed using the pooled standard deviation; positive values indicate higher scores in Profile 1 and negative values indicate higher scores in Profile 2. *p*-values are reported without correction for multiplicity given the exploratory and descriptive purpose of this table; the primary inferential analyses are reported in the main manuscript. Bold *p*-values indicate statistically significant differences at *p* < .05.

**Table 2:** Demographic and clinical characteristics of the full analytical sample and by latent profile membership ( *N*= 306).

Variable	Full Sample (*N* = 306)	Profile 1 (*n* = 212) *M (SD)*	Profile 2 (*n* = 94) *M (SD)*	Effect Size	*p*
Age (years)	57.65 (11.83)	56.75 (11.73)	59.67 (11.86)	*d* = −0.248	.160
Baseline MDD Severity	0.62 (1.69)	0.51 (1.52)	0.86 (2.00)	*d* = −0.209	.326
Chronic conditions (past 12 months)	2.08 (1.99)	1.72 (1.68)	2.87 (2.37)	*d* = −0.600	< .001
Medications (past 30 days)	5.21 (18.84)	4.83 (18.54)	6.05 (19.56)	*d* = −0.065	.663
Supplements (past 30 days)	1.82 (2.00)	1.91 (2.05)	1.61 (1.9)	*d* = 0.152	.417
Psychiatric diagnoses (count)	0.17 (0.46)	0.16 (0.46)	0.19 (0.47)	*d* = −0.067	.663
SUD severity	78.42 (14.71)	80.01 (12.1)	74.81 (18.94)	*d* = 0.358	0.078
Sex	Male: 134 (43.8%) Female: 172 (56.2%)	Male: 88 (41.5%) Female: 124 (58.5%)	Male: 46 (48.9%) Female: 48 (51.1%)	V = 0.062	.465
Race/Ethnicity	Multiracial: 2 (0.7%) White: 288 (96%) African American: 6 (2%) Native American: 1 (0.3%) Asian: 1 (0.3%) Other: 2 (0.7%)	Multiracial: 2 (1%) White: 202 (96.2%) African American: 3 (1.4%) Native American: 1 (0.5%) Asian: 1 (0.5%) Other: 1 (0.5%)	Multiracial: 0 (0%) White: 86 (95.6%) African American: 3 (3.3%) Native American: 0 (0%) Asian: 0 (0%) Other: 1 (1.1%)	V = 0.104	.663
Education Level	No high school degree: 15 (4.9%) High school: 82 (26.8%) Some college: 81 (26.5%) College education and above: 128 (41.8%)	No high school degree: 8 (3.8%) High school: 56 (26.4%) Some college: 54 (25.5%) College education and above: 94 (44.3%)	No high school degree: 7 (7.4%) High school: 26 (27.7%) Some college: 27 (28.7%) College education and above: 34 (36.2%)	V = 0.101	.532

*Note*. MDD = major depressive disorder; SUD = substance use disorder. *d* = Cohen's *d*; *M* = mean; *N* = total sample size; *n* = subgroup sample size; *p* = probability value; *SD* = standard deviation; V = Cramér's V. Continuous variables are presented as *M (SD)*; categorical variables are presented as *n* (%). Demographic and clinical variables were not included as latent profile analysis indicators; between-profile comparisons are presented here as post hoc characterizations of profile membership. Welch's independent-samples *t*-tests were used for continuous variables; Pearson's chi-square tests were used for categorical variables. Effect sizes for continuous variables are reported as Cohen's *d*, computed using pooled standard deviation; positive values indicate higher scores in Profile 1 and negative values indicate higher scores in Profile 2. Effect sizes for categorical variables are reported as Cramér's V (range: 0–1; higher = stronger association). -values are unadjusted; no statistically significant between-profile differences were observed for any demographic or clinical variable except chronic conditions (*p* < .001), indicating that the profiles did not differ systematically on background characteristics.

### 3.2 Sample attributes and selection bias

Before the LPA classification, baseline descriptive statistics for all 42 original study variables in the full analytic sample (*N* = 306) are reported (Table S3). Compared with excluded participants, included participants showed significantly healthier actigraphy-based sleep indices (|*d*| = 0.19–0.42), higher MSD IL-8 levels (*d* = 0.26), lower ICAM-1 and IL-10 levels (*d* = −0.31 to −0.14), and lower SUD severity (*d* = −0.13) (all *p* values ≤ .002). However, they did not differ significantly in baseline MDD severity (*d* = −0.05, *p* = .610).

### 3.3 LPA model selection

The 2-profile VII (variable volume, spherical covariance) solution was chosen based on consistent AIC, BIC, entropy (E = .842), and mean posterior classification probability (.940; Table S7). Furthermore, this solution yielded excellent split-sample replication stability (average ARI = .948; Table S6). In contrast, solutions with three or more profiles displayed worse replication stability and were therefore rejected. Finally, the 13-variable set provided more parsimony than the original 42-variable set (Table S7).

### 3.4 Profile characteristics

Profile 1 (*n* = 212; 69.3%) was marked by sufficient sleep and low systemic proinflammatory activity, whereas Profile 2 (*n* = 94; 30.7%) was characterized by fragmented sleep and greater proinflammatory activity (all *p* values ≤ .024; Table 1). Notably, the strongest effect sizes were observed for sleep efficiency (Profile 1: *M* = 85.22, *SD* = 4.99; Profile 2: *M* = 75.38, *SD* = 10.88; *d* = 1.35), sleep onset latency (Profile 1: *M* = 18.04, *SD* = 12.65; Profile 2: *M* = 40.95, *SD* = 29.60; *d* = −1.18), ELISA IL-6 (Profile 1: *M* = 1.05, *SD* = 0.35; Profile 2: *M* = 1.56, *SD* = 0.53; *d* = −1.24), and MSD IL-6 (Profile 1: *M* = 0.56, *SD* = 0.19; Profile 2: *M* = 0.97, *SD* = 0.51; *d* = −1.25).

### 3.5 Profile membership predictive value for 9-year MDD severity

**Fig. 2:**
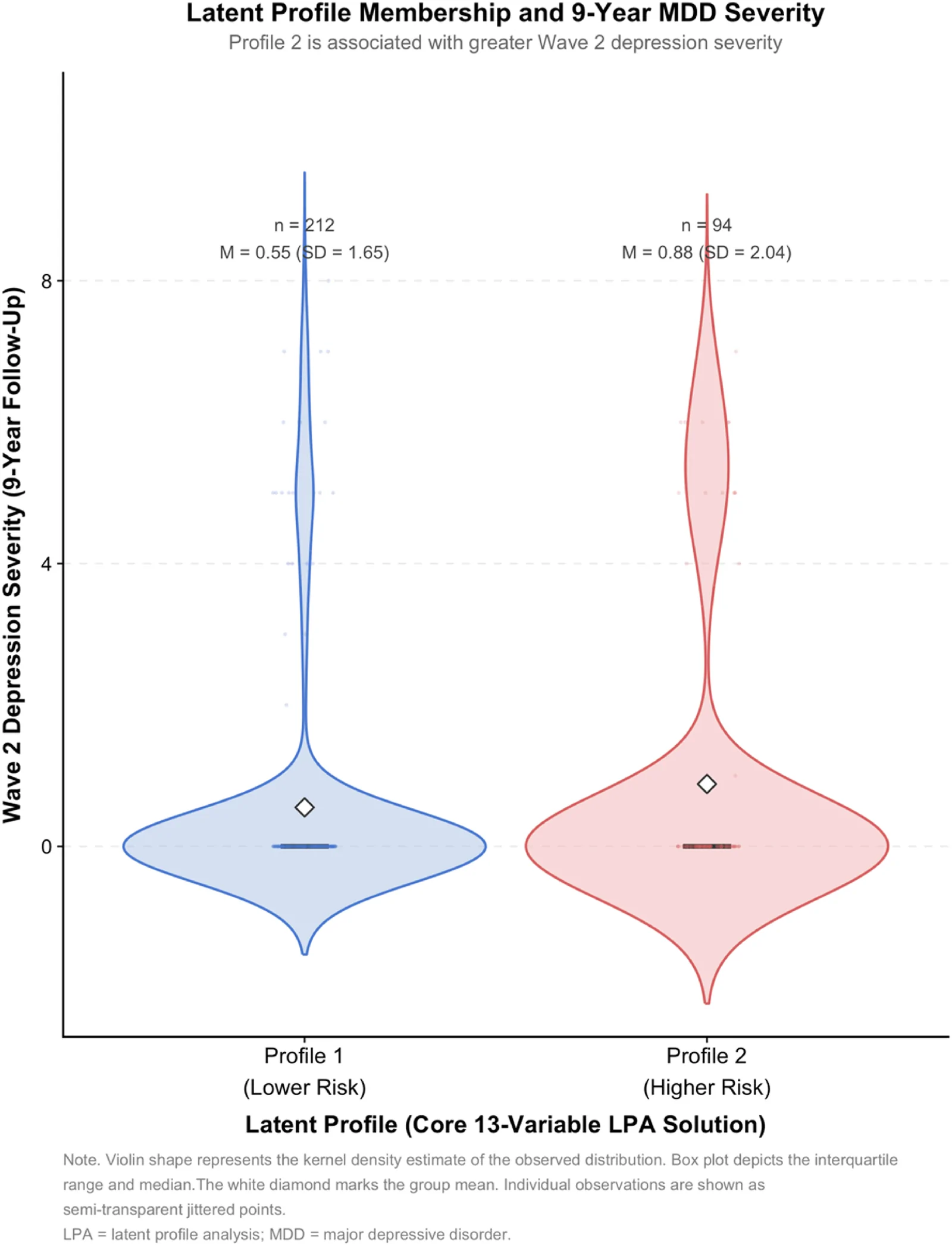
Wave 2 depression severity at 9-year follow-up by latent profile membership. *Note*. LPA = latent profile analysis; MDD = major depressive disorder. Violin plots display the kernel density estimate of the observed distribution for each latent profile derived from the core 13-variable latent profile analysis (LPA) solution. Embedded box plots depict the interquartile range and median; the open diamond (◇) marks the group mean. Individual observations are shown as semi-transparent jittered points. Profile 1 (*n* = 212, *M* = 0.55, *SD* = 1.65) represents the lower-risk group; Profile 2 (*n* = 94, *M* = 0.88, *SD* = 2.04) represents the higher-risk group. Profile 2 membership was associated with greater Wave 2 major depressive disorder severity at the 9-year follow-up.

Fig. 2 visualizes 9-year depression severity by profile membership. Table S12 presents the multiple regression of the LPA indicators predicting 9-year MDD severity. However, the model fit of this 13-indicator, variable-centered regression (AIC = 1203.99, BIC = 1323.15) was not as good as that of the regression examining how well the 2-profile membership predicted 9-year MDD severity (AIC = 1192.54, BIC = 1323.15 1267.01). Afterward, Table S13 presents the sequential regression model examining the profile membership model predicting 9-year MDD severity. In Model 1 (unadjusted), Profile 2 membership was significantly linked to higher 9-year MDD severity compared with Profile 1 (*d* = 0.448, *p* < .001). This relationship remained significant after controlling for demographics (Model 2: *d* = 0.401, *p* = .001) and persisted even after adjusting for MDD severity (Model 3: *d* = 0.244, *p* = .039), suggesting that it was not attributable to baseline MDD severity. In the completely adjusted model comprised of clinical covariates (e.g., psychiatric diagnostic counts and SUD severity), despite attenuation to statistical non-significance, the effect size remained practically significant (*d* = 0.208, *p* = .079; Table 3).

**Table 3:** Multiple regression estimates for latent profile membership predicting wave 2 MDD severity nine years later, with demographic covariates and baseline MDD severity (model 5) ( *N*= 306).

Model 5 (Full Model + Clinical Covariates)	*b*	*(SE)*	*t*	*p*	Cohen's *d*
Intercept	3.512	(0.996)	3.527	< .001	0.416
Profile 2 vs. Profile 1	0.396	(0.225)	1.765	.079	0.208
Age (years)	−0.005	(0.008)	−0.581	.562	−0.069
Women vs. women	−0.176	(0.206)	−0.855	.393	−0.101
Multiracial	−1.567	(1.361)	−1.152	.250	−0.136
White	−2.441	(0.699)	−3.494	.001	−0.412
African American	−3.570	(0.966)	−3.696	< .001	−0.436
Native American or Aleutian Islander/Eskimo	−3.434	(1.828)	−1.879	.061	−0.222
Asian or Pacific Islander	−3.854	(1.822)	−2.115	.035	−0.250
Other	−2.964	(1.373)	−2.159	.032	−0.255
College education vs. High school	0.027	(0.240)	0.114	.909	0.013
College education vs. No high school degree	−0.193	(0.462)	−0.419	.676	−0.049
College education vs. Some college	0.217	(0.240)	0.902	.368	0.106
Baseline MDD severity	0.090	(0.102)	0.884	.377	0.104
Chronic conditions (past 12 months)	0.038	(0.057)	0.666	.506	0.079
Medications (past 30 days)	−0.006	(0.005)	−1.125	.261	−0.133
Supplements (past 30 days)	0.117	(0.051)	2.291	.023	0.270
Psychiatric diagnoses (count)	0.708	(0.370)	1.914	.057	0.226
SUD severity	−0.009	(0.007)	−1.242	.215	−0.147

*Note*. *p < .05, ***p < .001. AIC = Akaike Information Criterion; BIC = Bayesian Information Criterion; MDD = major depressive disorder. β = unstandardized regression coefficient; *F* = *F*-statistic; *N* = total sample size; *p* = probability value; *R*^2^ = coefficient of determination. Unstandardized regression coefficients are presented with standard errors in parentheses. Model 1 enters latent profile membership (Profile 2 vs. Profile 1) as the sole predictor. Model 2 adds age (years) and sex (women vs. men) as demographic covariates. Model 3 additionally adjusts for baseline Wave 1 MDD severity to examine whether the profile effect persists after accounting for pre-existing depressive symptom load. A dash (−) indicates the predictor was not included in that model. The attenuation of the profile coefficient from Model 1 (β = 0.839) to Model 5 (β = 0.396) while retaining a meaningful effect size (Cohen's *d* = 0.208) indicates that latent profile membership predicts nine-year MDD severity over and above baseline depression and demographic factors. Race/ethnicity was excluded from this model to maintain parsimony; full demographic covariate estimates including race/ethnicity and education are reported separately. *p*-values are presented without adjustment for multiplicity.

### 3.6 Added value of latent profile analysis (LPA) and sensitivity analyses

Across all model fit evaluation statistics, LPA profile membership (Model 5) attained the lowest AIC and BIC values (AIC = 1192.54, BIC = 1267.01) compared with the 13-indicator, variable-centered regression model, three composite score models, K-means clustering, and LASSO regression models (Table S8). In addition to these fit advantages, LPA profile membership achieved comparable adjusted *R*^*2*^ values while maintaining stronger parsimony. Notably, the 13-indicator, variable-centered regression model showed that PSQI global score was the sole unique significant predictor of 9-year MDD severity (β = 0.075, *p* = .023; Table S12). Finally, results from the sensitivity analysis indicated that neither the additive nor the multiplicative model performed better than the LPA reference model on parsimony-adjusted fit (INT-B BIC = 1273.0 vs. Model 5 BIC = 1267.0; Table S11), further supporting the LPA's relative strength.

## 4 Discussion

In the present study, LPA detected two distinct profiles: Profile 1 (adequate sleep, low inflammation; 69.3%) and Profile 2 (increased proinflammatory activity, sleep fragmentation; 30.7%). Membership in Profile 2 forecasted higher nine-year MDD severity after adjusting for baseline depression and demographic factors. Consistent with prior research (Beattie et al., 2022; Moran et al., 2017), LPA offered superior model fit compared with variable-centered alternatives.

The analytic sample for the LPA differed from excluded participants primarily due to the use of actigraphy-based sleep data rather than differences in baseline depression severity, although included participants also showed small differences in selected inflammatory markers and substance use disorder severity. Most dropouts resulted from not following actigraphy procedures (Slyepchenko et al., 2023) rather than mental health or immune issues. The lack of group differences in baseline depression severity suggests the findings are not considered biased by high-risk selection. This dropout rate matches earlier MIDUS studies reporting higher dropout from activity monitor protocols than blood-based studies (Love et al., 2010; White et al., 2017). Thus, the profiles reflect middle-aged and older community adults with sleep and immune data, rather than a unique clinical group.

LPA identified two groups: Profile 1 (low sleep problems, low inflammation; 69.3%) and Profile 2 (high sleep problems, high inflammation; 30.7%), mirroring recent studies reporting similar biological patterns (Smith and Lee, 2022; Yip et al., 2021). Large differences in sleep efficiency, sleep onset latency, and IL-6 levels replicate earlier research linking disrupted sleep to increased inflammation in middle-aged women (Nowakowski et al., 2018). Profile 2 represents a clinically relevant risk group for depression, which would not emerge if examining sleep or inflammation separately (Irwin, 2025). The two-group finding displayed consistency across analytic versions and stability across split samples, strengthening the robustness of the results.

Profile 2 was associated with higher depression severity after 9 years, even when accounting for baseline depressive symptoms. The reduction in effect size from *d* = 0.448 to *d* = 0.244 after adjusting for baseline MDD severity suggests that earlier depression partially, but not fully, explains this association, with further attenuation to *d* = 0.208 once the full set of clinical covariates was added. Thus, the neuroimmune type emerged as a long-term risk factor for depression beyond baseline depressive symptoms (Dantzer et al., 2008; Raison and Miller, 2011). These findings align with longitudinal studies showing that sleep problems relate to increased inflammation and subsequent mental health issues (Ballesio, 2023; Cho et al., 2015). However, the 9-year interval introduces potential confounders, such as medication use, lifestyle changes (Kvam et al., 2016; Molero et al., 2025), or development of immune/metabolic diseases (Capuron and Miller, 2011), which may also contribute to depression. The observational design allowed examination of baseline neuroimmune types linked to later depression but did not clarify specific predictive relationships. Additional analyses indicated that sleep and inflammation did not interact synergistically; the “two-hit” concept of depression refers to shared risk factors rather than a true interaction (Irwin, 2025).

In this study, LPA provided a better fit than 13 separate indicators in regression, summary scores, K-means clustering, or LASSO regression, suggesting that profile grouping offers more value than standard methods (Bauer and Shanahan, 2007; Spurk et al., 2020). Only the PSQI Global Score of past-month sleep disturbances predicted depression 9 years later among single variables, and no immune marker alone was a significant predictor, emphasizing the importance of combined sleep and inflammation measures (Nusslock et al., 2024; Yin et al., 2023). These results point to potential shared biological drivers for inflammation and sleep (Besedovsky et al., 2019). The autonomic nervous system and HPA axis influence both proinflammatory activity and sleep continuity; thus, dysfunction in these systems could contribute to Profile 2 (fragmented sleep, increased inflammation) (Irwin et al., 2016). Recent research also showed that gut bacteria can alter sleep and inflammation via neurochemical and neural pathways (Sejbuk et al., 2024). Although gut microbiome was not measured here, this framework situates Profile 2 within a broader brain–immune–gut system. Future studies should measure the nervous system, stress, and gut microbiome concurrently to test these hypotheses.

The present study had several limitations. First, neural sleep indicators and polysomnography biomarkers were not examined and may differ from actigraphy-based estimates, clinical interviews, and self-reports used here (Danzig et al., 2020; Liguori et al., 2023) and could act as confounders. Future research should investigate neural indicators of sleep quality in concert with inflammatory markers to determine if they improve the model predictions of MDD severity. Prospective, multimodal methods (e.g., MRI) may clarify how fragmented sleep and high inflammation affect monoamine activity and alter future MDD symptoms (Batail et al., 2025; Saggu et al., 2025). Including neurocognitive correlates (e.g., executive dysfunction) in prediction models could further elucidate their interplay with inflammation and sleep in predicting long-term MDD symptoms. Second, sleep disturbances and proinflammatory activity might both have bidirectional links with MDD severity (Mac Giollabhui et al., 2021). The focus here was on prognostic value for long-term MDD prediction, but future studies should examine the mechanisms underlying the bidirectional links between sleep and MDD, and inflammation and MDD, in greater detail. Third, inferences were made at the between-person rather than within-person level, risking ecological fallacy (Saqr et al., 2026). More prospective research with at least three assessments is needed to clarify within-person effects and enhance evidence for scalable treatments. Fourth, the single nine-year interval limits conclusions about the profile's predictive value over longer periods, such as 20 years. Finally, the sample was predominantly White; future work should include more culturally-diverse populations to assess generalizability.

Despite these limitations, some strengths were noteworthy. First, an advanced approach using LPA identified two clearly defined profiles, or subtypes, with prognostic value for predicting future MDD symptoms. Second, the effect sizes obtained suggested that the findings were clinically meaningful or practically significant. Lastly, the predictive value of profile membership was independent of baseline MDD symptoms and important demographic confounders.

Initial neuroimmune risk classification thresholds, based on well-established empirical and clinical benchmarks, are shown in Table S10 and await external validation and replication across culturally diverse samples. If replicated, several clinical translational implications merit attention. Early detection of the Profile 2 subtype–characterized by fragmented sleep and high proinflammatory activity–might facilitate risk-stratified, precision-based prevention before MDD onset or recurrence (Scala et al., 2023). Scalable risk identification procedures that combine actigraphy-derived and proinflammatory markers may enable targeted prevention and treatment in community mental health and primary care settings (Rykov et al., 2021). Individualized lifestyle modification interventions that promote anti-inflammatory activities and sleep continuity, based on cognitive-behavioral therapy for insomnia, regular physical activity, and the Mediterranean diet, can be beneficial for this subtype (Lassale et al., 2019). Concurrent immune-regulating approaches merit systematic investigation among persons who resemble Profile 2 (Kappelmann et al., 2018). Together, these strategies should encompass the principles of precision psychiatry by providing evidence-based, scalable treatments toward biologically based, high-risk profiles (Kas et al., 2025).

## Funding

The present study was funded by the 10.13039/501100001352National University of Singapore Presidential Young Professorship (PYP) Start-Up Grant and the PYP White Space Fund, both awarded to Nur Hani Zainal (A-0009981-00-00; A-0009981-01-00). Since 1995, the Midlife Development in the United States (MIDUS) study has been funded by the following sources: the John D. and Catherine T. MacArthur Foundation Research Network and the National Institute on Aging (P01-AG020166; U19-AG051426). All funding agencies are not responsible for the analyses or interpretations presented here. The contents do not represent the views of the U.S. Department of Veterans Affairs or the United States Government.

## CRediT authorship contribution statement

**Nur Hani Zainal:** Conceptualization, Data curation, Formal analysis, Funding acquisition, Investigation, Methodology, Resources, Software, Supervision, Validation, Visualization, Writing – original draft, Writing – review & editing. **Sherry A. Beaudreau:** Conceptualization, Data curation, Formal analysis, Investigation, Methodology, Supervision, Validation, Visualization, Writing – original draft, Writing – review & editing.

## Declaration of competing interests

The authors declare that they have no known competing financial interests or personal relationships that could have appeared to influence the work reported in this paper.

## Data Availability

The authors do not have permission to share data.
