# Supplementary material for: Sleep fragmentation and heightened inflammation: A latent profile subtype predicting nine-year major depressive disorder severity in the MIDUS study
Source: Brain Behav Immun Health. 2026 Aug 11;56:101332. doi: 10.1016/j.bbih.2026.101332 (PMC13543093; doi:10.1016/j.bbih.2026.101332)
Supplement: Supplementary file 1 [file mmc1.docx]

# Online Supplemental Materials (OSM)

## Table S1

*Assay Platforms, Reagent Kits, Catalog Numbers, and Manufacturers for Inflammatory Biomarkers in the MIDUS Biomarker Project*

| **Biomarker** | **Assay Platform** | **Kit/ Reagent Name** | **Catalog Number** | **Manufacturer** | **Manufacturer Location** | **Analyte Class** | **Sample Matrix** | **Laboratory** |
| --- | --- | --- | --- | --- | --- | --- | --- | --- |
| Interleukin-6 (IL-6) | ELISA | Quantikine® High-Sensitivity Human IL-6 Immunoassay | HS600B | R&D Systems (Bio-Techne) | Minneapolis, MN, USA | Pro-inflammatory cytokine | Serum | MIDUS Biocore Laboratory, University of Wisconsin-Madison, Madison, WI |
| Soluble E-Selectin (sE-Selectin/ CD62E) | ELISA | Parameter™ Human sE-Selectin Immunoassay | SSLE00 | R&D Systems (Bio-Techne) | Minneapolis, MN, USA | Cell adhesion molecule | Serum | Laboratory for Clinical Biochemistry Research, University of Vermont, Burlington, VT |
| Soluble ICAM-1 (sICAM-1/ CD54) | ELISA | Quantikine® Human sICAM-1 Immunoassay | SCD540 | R&D Systems (Bio-Techne) | Minneapolis, MN, USA | Cell adhesion molecule | Serum | Laboratory for Clinical Biochemistry Research, University of Vermont, Burlington, VT |
| C-Reactive Protein (CRP) | Immunonephelometry (BNII Nephelometer) | Particle-Enhanced Immunonephelometric Assay (PENIA); undetectable samples re-assayed with MSD high-sensitivity kit | Not Applicable (N/A) (platform-based assay) | Dade Behring Inc. (now Siemens Healthineers) | Deerfield, IL, USA | Acute-phase protein | Citrated plasma | Laboratory for Clinical Biochemistry Research, University of Vermont, Burlington, VT |
| Fibrinogen | Immunonephelometry (BNII Nephelometer) | N Antiserum to Human Fibrinogen | N/A (platform-based assay) | Dade Behring Inc. (now Siemens Healthineers) | Deerfield, IL, USA | Acute-phase protein/ coagulation factor | Citrated plasma | Laboratory for Clinical Biochemistry Research, University of Vermont, Burlington, VT |
| Interleukin-6 (IL-6) – MSD platform | Electrochemiluminescence (ECL) | V-PLEX Custom Human Cytokine Kit | K151A0H-2 | Meso Scale Diagnostics (MSD) | Rockville, MD, USA | Pro-inflammatory cytokine | Serum | Laboratory for Clinical Biochemistry Research, University of Vermont, Burlington, VT |
| Interleukin-8 (IL-8/ CXCL8) | Electrochemiluminescence (ECL) | V-PLEX Custom Human Cytokine Kit | K151A0H-2 | Meso Scale Diagnostics (MSD) | Rockville, MD, USA | Pro-inflammatory chemokine | Serum | Laboratory for Clinical Biochemistry Research, University of Vermont, Burlington, VT |
| Interleukin-10 (IL-10) | Electrochemiluminescence (ECL) | V-PLEX Custom Human Cytokine Kit | K151A0H-2 | Meso Scale Diagnostics (MSD) | Rockville, MD, USA | Anti-inflammatory cytokine | Serum | Laboratory for Clinical Biochemistry Research, University of Vermont, Burlington, VT |
| Tumor Necrosis Factor-alpha (TNF-α) | Electrochemiluminescence (ECL) | V-PLEX Custom Human Cytokine Kit | K151A0H-2 | Meso Scale Diagnostics (MSD) | Rockville, MD, USA | Pro-inflammatory cytokine | Serum | Laboratory for Clinical Biochemistry Research, University of Vermont, Burlington, VT |

*Note*. ELISA = enzyme-linked immunosorbent assay; ECL = electrochemiluminescence; ICAM-1 = Intercellular Adhesion Molecule-1; IL = interleukin; MSD = Meso Scale Diagnostics; PENIA = particle-enhanced immunonephelometric assay; TNF-α = tumor necrosis factor-alpha. All biomarkers were collected as part of the Midlife in the United States (MIDUS) Biomarker Project and analyzed from blood specimens collected following an overnight fast, centrifuged, and stored at −70°C prior to shipment on dry ice. Serum was used for all assays except C-reactive protein and fibrinogen, which were measured from citrated plasma. The V-PLEX Custom Human Cytokine Kit (Cat. No. K151A0H-2; Meso Scale Diagnostics, Rockville, MD, USA) simultaneously quantified IL-6, IL-8, IL-10, and TNF-α in a single multiplex panel using electrochemiluminescence detection. C-reactive protein samples falling below the detection limit of the BNII Nephelometer were re-assayed using a Meso Scale Diagnostics high-sensitivity kit. Inflammatory marker assays were conducted at the Laboratory for Clinical Biochemistry Research, University of Vermont, Burlington, VT, with the exception of IL-6 (ELISA), which was conducted at the MIDUS Biocore Laboratory, University of Wisconsin–Madison, Madison, WI. Assay details are consistent with those reported in Love et al. (2010).

## Table S2

*Shapiro–Wilk Tests of Normality for Key Sleep Variables Used in the Latent Profile Analysis (n = 306)*

| **Variable** | **Skewness** | **Kurtosis** | **W** | ***p*** | **Non-Normality** |
| --- | --- | --- | --- | --- | --- |
| Sleep efficiency (SE) | -1.629 | 3.492 | 0.870 | .000 | Yes |
| Wake after sleep onset (WASO) | 1.142 | 2.385 | 0.931 | .000 | Yes |
| Sleep onset latency (SOL) | 1.902 | 3.866 | 0.801 | .000 | Yes |
| PSQI Global Score | 1.035 | 0.862 | 0.916 | .000 | Yes |

*Note*. p = probability value; PSQI = Pittsburgh Sleep Quality Index; SE = sleep efficiency; SOL = sleep onset latency; W = Shapiro-Wilk statistic; WASO = wake after sleep onset. The Shapiro–Wilk test was selected over the Kolmogorov-Smirnov test given its superior statistical power for detecting departures from normality in samples of this size (Mohd Razali & Yap, 2011). W is the Shapiro–Wilk test statistic, with values closer to 1.00 indicating greater conformity to a normal distribution. Non-normality was defined as *p* < .05. All four variables exhibited statistically significant departures from normality, consistent with commonly observed distributional properties of actigraphy and subjective sleep data (Harvey, 2008). Log-transformed biomarker variables were assessed separately and are reported in the biomarker correlation matrix. These distributional findings support the use of Welch’s independent-samples t-tests (which do not assume normality of the outcome variable) for between-profile comparisons, and the use of Gaussian mixture models within the latent profile analysis framework (which accommodate non-normal marginal distributions via mixture decomposition).

## Table S3

*Comparison of Baseline Characteristics Between Included and Excluded Participants Across Actigraphy Sleep, Inflammatory Biomarker, Self-Report, and Clinical Variables*

| **Group** |  | **Included (*n* = 306)** | |  | **Excluded (*n* = 748)** | |  | **Effect Size** | |
| --- | --- | --- | --- | --- | --- | --- | --- | --- | --- |
| **Variable** |  | ***M*** | ***(SD)*** |  | ***M*** | ***(SD)*** |  | ***d*** | ***p (B-H)*** |
| Total Activity Count (TAC) |  | 7585.72 | (4213.93) |  | 10995.05 | (9877.05) |  | -0.40 | < .001 |
| Activity Count Mean (ACM) |  | 17.64 | (9.88) |  | 25.12 | (22.15) |  | -0.39 | < .001 |
| Maximum Activity Count (MAC) |  | 510.93 | (162.43) |  | 555.93 | (257.44) |  | -0.19 | .002 |
| Onset Latency (OL) |  | 25.03 | (22.04) |  | 33.47 | (30.49) |  | -0.30 | < .001 |
| Snooze Time (SNT) |  | 13.37 | (14.48) |  | 19.39 | (19.96) |  | -0.33 | < .001 |
| Sleep Efficiency (EFF) |  | 82.20 | (8.58) |  | 77.61 | (16.27) |  | 0.32 | < .001 |
| Wake After Sleep Onset (WASO) |  | 44.28 | (19.79) |  | 60.43 | (47.20) |  | -0.39 | < .001 |
| Wake Time (WT) |  | 44.26 | (19.78) |  | 60.91 | (47.86) |  | -0.40 | < .001 |
| Percent Wake (PW) |  | 10.37 | (4.73) |  | 14.40 | (11.34) |  | -0.41 | < .001 |
| Wake Bouts (WB) |  | 31.82 | (10.57) |  | 37.03 | (18.77) |  | -0.31 | < .001 |
| Average Wake Bout (AWB) |  | 1.40 | (0.47) |  | 1.65 | (0.90) |  | -0.30 | < .001 |
| Sleep Time (SLT) |  | 381.54 | (62.28) |  | 349.98 | (93.14) |  | 0.37 | < .001 |
| Percent Sleep (PSL) |  | 89.64 | (4.73) |  | 85.44 | (11.55) |  | 0.42 | < .001 |
| Sleep Bouts (SLB) |  | 32.12 | (10.58) |  | 37.93 | (19.86) |  | -0.33 | < .001 |
| Average Sleep Bout (ASB) |  | 14.46 | (7.49) |  | 16.88 | (13.42) |  | -0.20 | .001 |
| MDD Severity |  | 0.68 | (1.88) |  | 0.78 | (2.08) |  | -0.05 | .610 |
| GAD Severity |  | 12.75 | (5.99) |  | 13.14 | (6.39) |  | -0.06 | .493 |
| Panic Disorder Severity |  | 0.38 | (1.12) |  | 0.45 | (1.20) |  | -0.06 | .493 |
| MSD IL-6 Levels |  | 0.69 | (0.38) |  | 0.71 | (0.66) |  | -0.04 | .610 |
| MSD IL-8 Levels |  | 2.73 | (0.36) |  | 2.59 | (0.58) |  | 0.26 | < .001 |
| MSD IL-10 Levels |  | 0.25 | (0.19) |  | 0.31 | (0.51) |  | -0.14 | .008 |
| MSD TNF-α Levels |  | 1.16 | (0.22) |  | 1.17 | (0.31) |  | -0.03 | .709 |
| ELISA IL-6 Levels |  | 1.20 | (0.48) |  | 1.20 | (0.56) |  | 0.01 | .872 |
| ELISA Fibrinogen levels |  | 5.80 | (0.25) |  | 5.83 | (0.29) |  | -0.11 | .166 |
| ELISA CRP Levels |  | 1.05 | (0.71) |  | 1.06 | (0.79) |  | -0.01 | .872 |
| ELISA E-Selectin Levels |  | 3.70 | (0.50) |  | 3.64 | (0.52) |  | 0.11 | .180 |
| ELISA ICAM-1 Levels |  | 5.55 | (0.32) |  | 5.66 | (0.36) |  | -0.31 | < .001 |
| Menopausal Drug Use Counts |  | 0.68 | (0.89) |  | 0.78 | (0.96) |  | -0.11 | .189 |
| Menstrual Symptom Counts |  | 1.16 | (0.67) |  | 1.14 | (0.67) |  | 0.03 | .764 |
| Aging Worries Count |  | 5.30 | (1.68) |  | 5.49 | (1.73) |  | -0.11 | .189 |
| PSQI 1 (Subjective Sleep Quality) |  | 0.97 | (0.66) |  | 0.97 | (0.68) |  | 0.01 | .895 |
| PSQI 2 (Sleep Latency) |  | 0.91 | (0.90) |  | 0.87 | (0.93) |  | 0.05 | .610 |
| PSQI 3 (Sleep Duration) |  | 0.79 | (0.78) |  | 0.78 | (0.74) |  | 0.02 | .838 |
| PSQI 4 (Habitual Sleep Efficiency) |  | 0.56 | (1.00) |  | 0.61 | (0.99) |  | -0.05 | .631 |
| PSQI 5 (Sleep Disturbances) |  | 1.27 | (0.54) |  | 1.29 | (0.57) |  | -0.03 | .714 |
| PSQI 6 (Use of Sleeping Medication) |  | 0.50 | (1.00) |  | 0.60 | (1.09) |  | -0.10 | .208 |
| PSQI 7 (Daytime Dysfunction) |  | 0.83 | (0.66) |  | 0.80 | (0.67) |  | 0.05 | .610 |
| Psychiatric diagnoses (count) |  | 0.17 | (0.46) |  | 0.22 | (0.54) |  | -0.09 | .244 |
| SUD severity |  | 22.62 | (1.86) |  | 23.10 | (4.21) |  | -0.13 | .027 |
| Chronic conditions (past 12 months) |  | 2.02 | (1.89) |  | 2.27 | (2.31) |  | -0.11 | .144 |
| Medications (past 30 days) |  | 1.39 | (1.49) |  | 1.41 | (1.50) |  | -0.01 | .872 |
| Supplements (past 30 days) |  | 1.82 | (2.01) |  | 2.06 | (2.01) |  | -0.12 | .144 |

*Note*. ACM = activity count mean; ASB = average sleep bout; AWB = average wake bout; CRP = C-reactive protein; EFF = sleep efficiency; ELISA = enzyme-linked immunosorbent assay; GAD = generalized anxiety disorder; ICAM-1 = Intercellular Adhesion Molecule-1; IL = interleukin; LPA = latent profile analysis; MAC = maximum activity count; MDD = major depressive disorder; MIDUS = Midlife in the United States; MSD = Meso Scale Diagnostics; OL = onset latency; PSL = percent sleep; PSQI = Pittsburgh Sleep Quality Index; PW = percent wake; SLB = sleep bouts; SLT = sleep time; SNT = snooze time; SUD = substance use disorder; TAC = total activity count; TNF-α = tumor necrosis factor-alpha; WASO = wake after sleep onset; WB = wake bouts; WT = wake time. *M* = mean; *SD* = standard deviation; *d* = Cohen’s d; *p* (B-H) = Benjamini–Hochberg adjusted *p*-value. The included sample (*n* = 306) comprised participants with complete data on all latent profile analysis (LPA) indicator variables. The excluded sample (*n* = 748) comprised all remaining MIDUS Biomarker Project participants who did not meet this criterion. All biomarker variables are log-transformed prior to analysis. Welch’s independent-samples t-tests were used for all continuous variable comparisons. Effect sizes are reported as Cohen’s d, with positive values indicating higher scores in the included group and negative values indicating higher scores in the excluded group. p-values were adjusted for multiple comparisons using the Benjamini-Hochberg false discovery rate procedure across all 42 variables. Bold *p*-values indicate statistically significant differences at *p* < .05 (BH-adjusted).

## Table S4

*Pearson Correlations Among Log-Transformed Inflammatory Biomarkers in the Analytical Sample (n = 306)*

| **Variable** | **1** | **2** | **3** | **4** | **5** | **6** | **7** | **8** | **9** |
| --- | --- | --- | --- | --- | --- | --- | --- | --- | --- |
| 1. Log CRP | 1.000 | .508 | .326 | .237 | .491 | .246 | .477 | .121 | .115 |
| 2. Log IL-6 | .508 | 1.000 | .379 | .182 | .398 | .119 | .853 | .101 | .115 |
| 3. Log TNF-α | .326 | .379 | 1.000 | .083 | .167 | .381 | .367 | .304 | .322 |
| 4. Log E-Selectin | .237 | .182 | .083 | 1.000 | .088 | .078 | .080 | .051 | .026 |
| 5. Log Fibrinogen | .491 | .398 | .167 | .088 | 1.000 | .014 | .313 | .094 | .084 |
| 6. Log ICAM-1 | .246 | .119 | .381 | .078 | .014 | 1.000 | .224 | .181 | .126 |
| 7. Log MSD IL-6 | .477 | .853 | .367 | .080 | .313 | .224 | 1.000 | .141 | .093 |
| 8. Log MSD IL-8 | .121 | .101 | .304 | .051 | .094 | .181 | .141 | 1.000 | .248 |
| 9. Log MSD IL-10 | .115 | .115 | .322 | .026 | .084 | .126 | .093 | .248 | 1.000 |

*Note*. CRP = C-reactive protein; ELISA = enzyme-linked immunosorbent assay; ICAM-1 = Intercellular Adhesion Molecule-1; IL = interleukin; MSD = Meso Scale Diagnostics; TNF-α = tumor necrosis factor-alpha. All biomarker values were log-transformed prior to analysis to address positive skewness. The correlation matrix is symmetric; values above and below the diagonal are identical and are presented in full for readability. Log ELISA IL-6 (Variable 2) and Log MSD IL-6 (Variable 7) reflect the same analyte (interleukin-6) measured via two distinct assay platforms–enzyme-linked immunosorbent assay (ELISA; R&D Systems, Cat. No. HS600B) and Meso Scale Diagnostics electrochemiluminescence (MSD ECL; V-PLEX Custom Human Cytokine Kit, Cat. No. K151A0H-2), respectively–and are retained as separate variables given their methodological non-equivalence. The notably high correlation between Log IL-6 and Log MSD IL-6 (*r* = .853) is expected given shared analyte identity.

## Table S5

*Number of Free Parameters and Cases-to-Parameters Ratios for EII, VII, and EEI Model Structures Across 2- to 6-Profile Solutions (n = 306)*

| **Number of Profiles** | **EII Model** | **VII Parameters** | **EEI Parameters** | **Cases Per Parameters EII** | **Cases Per Parameters VII** | **Cases Per Parameters EEI** |
| --- | --- | --- | --- | --- | --- | --- |
| 2 | 9 | 10 | 12 | 34.0 | 30.6 | 25.5 |
| 3 | 13 | 15 | 16 | 23.5 | 20.4 | 19.1 |
| 4 | 17 | 20 | 20 | 18.0 | 15.3 | 15.3 |
| 5 | 21 | 25 | 24 | 14.6 | 12.2 | 12.8 |
| 6 | 25 | 30 | 28 | 12.2 | 10.2 | 10.9 |

*Note*. EEI = equal volume, equal shape, and identity orientation (diagonal, equal covariance); EII = equal volume, identity shape, and identity orientation (spherical, equal covariance); VII = variable volume, identity shape, and identity orientation (spherical, unequal covariance). The row header (2–6) indicates the number of latent profiles. Parameter counts reflect the total number of free parameters estimated by each model structure for a given profile solution using 13 indicator variables. Cases-to-parameters ratios are computed as N ÷ number of free parameters for the respective model. A minimum cases-to-parameters ratio of 10:1 is recommended for stable latent profile solutions (Muthén & Muthén, 2002; Nylund-Gibson & Choi, 2018). Ratios falling below 10:1 are indicative of potential model instability and overfitting.

## Table S6

*Split-Sample Replication Stability of the VII Model Across 2- to 6-Profile Solutions Using the Adjusted Rand Index (n = 306)*

| **Number of profiles** | **Model** | **ARI Half 1** | **ARI Half 2** | **Mean ARI** | **Stable** | **Assessment** |
| --- | --- | --- | --- | --- | --- | --- |
| 2 | VII | 0.896 | 1.000 | 0.948 | Yes | Excellent stability |
| 3 | VII | 0.205 | 0.397 | 0.301 | No | Poor stability |
| 4 | VII | 0.889 | 0.946 | 0.917 | Yes | Excellent stability |
| 5 | VII | 0.716 | 0.532 | 0.624 | No | Moderate stability |
| 6 | VII | 0.576 | 0.663 | 0.620 | No | Moderate stability |

Note. ARI = Adjusted Rand Index; VII = variable volume, identity shape, and identity orientation. Stability was assessed by randomly dividing the analytical sample into two equal halves and fitting the VII (variable volume, spherical covariance) model independently in each half. The Adjusted Rand Index (ARI) quantifies agreement between the profile classifications derived from each half, correcting for chance agreement (Hubert & Arabie, 1985). ARI values range from 0 to 1, with higher values indicating greater cross-replication stability. A solution was classified as “Stable” if both half-sample ARI values exceeded 0.80, indicating excellent replication; “Moderate stability” reflects mean ARI values between 0.60 and 0.79; “Poor stability” reflects mean ARI values below 0.60 (Steinley, 2004). The VII model was evaluated across all profile solutions as it demonstrated the best overall BIC performance among converging model structures. The 2-profile VII solution yielded the highest mean ARI (0.948), supporting its selection as the optimal and most replicable solution.

## Table S7

*Robustness Comparison of the 13-Variable Core Set Versus the Original 43-Variable Set Across Latent Profile Solutions (2–6 Profiles) (n = 306)*

| **Number of Profiles** | **2** | **3** | **4** | **5** | **6** |
| --- | --- | --- | --- | --- | --- |
| Entropy winner | Tie | Tie | Tie | Tie | Tie |
| Entropy Core 13 | 0.842 | 0.831 | 0.781 | 0.781 | 0.799 |
| Entropy Original 43 | 0.842 | 0.831 | 0.781 | 0.781 | 0.799 |
| Average PP winner | Tie | Tie | Tie | Tie | Tie |
| Average PP Core 13 | 0.940 | 0.897 | 0.845 | 0.810 | 0.837 |
| Average PP Original 43 | 0.940 | 0.897 | 0.845 | 0.810 | 0.837 |
| Balance winner | Tie | Tie | Tie | Tie | Tie |
| Balance Core 13 Percentage | 31.700 | 19.610 | 19.610 | 16.990 | 10.460 |
| Balance Original 43 Percentage | 31.700 | 19.610 | 19.610 | 16.990 | 10.460 |
| ARI winner | Core 13 | Core 13 | Original 43 | Core 13 | – |
| ARI Core 13 | 0.897 | 0.629 | 0.601 | 0.554 | – |
| ARI Original 43 | 0.867 | 0.495 | 0.603 | 0.404 | – |
| Parsimony winner | Tie | Tie | Tie | Tie | Tie |
| Parsimony Core 13 | 0.308 | 0.231 | 0.154 | 0.154 | 0.154 |
| Parsimony Original 43 | 0.308 | 0.231 | 0.154 | 0.154 | 0.154 |
| Core 13 wins all | 1 | 1 | 0 | 1 | 0 |
| Original 43 wins all | 0 | 0 | 1 | 0 | 0 |
| Primary verdict | Core 13 more robust (primary metrics) | | | | |

Note. Each metric was computed separately for the Core 13-variable model and the Original 43-variable model across profile solutions ranging from 2 to 6. Entropy reflects classification certainty (range: 0–1; higher = better separation). Average posterior probability (PP) reflects the mean probability of correct class assignment (range: 0–1; higher = better). Balance reflects the percentage of cases assigned to the smallest profile, with higher values indicating more even profile distribution. The Adjusted Rand Index (ARI) reflects cross-validated classification agreement between two random split halves of the sample (range: 0–1; higher = greater stability; values below 0.60 indicate poor replication). Parsimony reflects the ratio of profiles to variables (range: 0–1; higher = more parsimonious). For each metric, a “winner” indicates which variable set performed better; “Tie” indicates equivalent performance. “Core 13 wins all” and “Original 43 wins all” are binary indicators (1 = that model outperformed on all primary metrics simultaneously; 0 = did not). ARI was not estimable (NA) for the 6-profile solution due to insufficient within-split sample sizes. The primary verdict was determined by majority wins across entropy, average PP, ARI, and balance. ARI = Adjusted Rand Index; NA = not applicable; PP = posterior probability.

## Table S8

*Predictive Performance of the Latent Profile Analysis Model Compared to Variable-Centered, Composite Score, K-Means, and LASSO Benchmark Approaches in Predicting Wave 2 MDD Severity (n = 306)*

| **Model** | ***R²*** | **Adjusted *R²*** | **AIC** | **BIC** |
| --- | --- | --- | --- | --- |
| LPA Model 5 (profile membership) | 20.26% | 15.26% | 1192.54 | 1267.01 |
| Model 6 (13 indicators, variable-centered) | 23.47% | 15.12% | 1203.99 | 1323.15 |
| C1: Sleep Composite | 20.71% | 15.74% | 1190.80 | 1265.28 |
| C2: Inflammation Composite | 19.65% | 14.61% | 1194.89 | 1269.36 |
| C3: Global Composite (all 13) | 19.97% | 14.95% | 1193.66 | 1268.13 |
| K-means (G = 2) | 19.59% | 14.54% | 1195.12 | 1269.59 |
| LASSO benchmark (CV R ≤ 0.0619) | 18.22% | – | – | – |

*Note*. AIC = Akaike Information Criterion; BIC = Bayesian Information Criterion; CV = cross-validation; LASSO = least absolute shrinkage and selection operator; LPA = latent profile analysis; MDD = major depressive disorder; *R²* = coefficient of determination. All models were evaluated on the same analytical sample (*n* = 306) using Wave 2 MDD severity as the outcome. LPA Model 5 (profile membership) uses latent profile assignment from the 2-profile solution as the sole predictor. Model 6 enters all 13 LPA indicator variables simultaneously in a variable-centered regression. C1 (Sleep Composite) uses the mean of the three actigraphy sleep indicators; C2 (Inflammation Composite) uses the mean of the nine log-transformed biomarker indicators; C3 (Global Composite) uses the mean of all 13 indicators. K-means (*G* = 2) uses cluster assignment from a 2-group *K*-means solution on the same 13 standardized variables as the primary predictor. The LASSO benchmark is a cross-validated LASSO regression across all 13 indicators; Adjusted *R²*, AIC, and BIC are not reported as they are not directly estimable under cross-validation. A dash (–) indicates the statistic is not applicable for the respective model. Lower AIC and BIC values indicate better model fit penalized for complexity. The comparable or superior Adjusted *R*² of LPA Model 5 relative to all alternative approaches, despite greater parsimony, supports the added value of the latent profile framework over variable-centered and composite alternatives.

## Table S9

*Sensitivity Analysis: Additive and Multiplicative Sleep × Inflammation Interaction Models Compared to the Latent Profile Analysis Reference Model in Predicting Wave 2 MDD Severity (n = 306)*

| **Model** | ***R²*** | **Adjusted *R²*** | **AIC** | **BIC** |
| --- | --- | --- | --- | --- |
| INT-A: Additive (sleep + inflammation) | 20.68% | 15.41% | 1193.0 | 1271.2 |
| INT-B: Multiplicative (+ sleep x inflammation interaction) | 21.67% | 16.17% | 1191.1 | 1273.0 |
| Model 5: LPA profile membership (reference) | 20.26% | 15.26% | 1192.5 | 1267.0 |

*Note*. AIC = Akaike Information Criterion; BIC = Bayesian Information Criterion; CRP = C-reactive protein; ICAM-1 = Intercellular Adhesion Molecule-1; IL = interleukin; LPA = latent profile analysis; MDD = major depressive disorder; MSD = Meso Scale Diagnostics; R² = coefficient of determination; SOL = sleep onset latency; TNF-α = tumor necrosis factor-alpha; WASO = wake after sleep onset. All models were estimated in the same analytical sample (*n* = 306) with Wave 2 MDD severity as the outcome. INT-A (additive) enters mean sleep composite (sleep efficiency, WASO, SOL) and mean inflammation composite (log-transformed CRP, IL-6, TNF-α, E-Selectin, Fibrinogen, ICAM-1, MSD IL-6, MSD IL-8, MSD IL-10) as separate continuous predictors. INT-B (multiplicative) adds a product term (sleep composite × inflammation composite) to INT-A to test whether the combined effect of sleep and inflammation on MDD severity exceeds the sum of their individual effects. Model 5 (LPA profile membership) enters the binary latent profile assignment (Profile 1 vs. Profile 2) as the sole predictor and serves as the reference model. Lower AIC values indicate better model fit penalized for the number of parameters; lower BIC values apply a stronger penalty for model complexity. The marginal improvement in Adjusted R² from INT-A to INT-B (15.41% vs. 16.17%) alongside higher BIC in INT-B (1273.0 vs. 1271.2) indicates that the multiplicative interaction does not meaningfully improve predictive performance over the additive model, and that Model 5 achieves comparable parsimony-adjusted fit.

## Table S10

*Clinical and Empirical Thresholds for Classifying Sleep Disturbance and Elevated Inflammatory Biomarker Levels Based on Published Reference Standards*

| **Variable** | **Threshold in log units** | **Direction** | **Unit** | **Label if exceeded** | **Label if normal** | **Citation** |
| --- | --- | --- | --- | --- | --- | --- |
| Sleep efficiency (SE) | 85.00 | Below | % | Poor sleep efficiency (< 85%) | Adequate sleep efficiency (≥ 85%) | Buysse et al. (2010); Ohayon et al. (2017) |
| Wake after sleep onset (WASO) | 30.00 | Above | minutes | Clinically elevated WASO (> 30 min) | Normal WASO (≤ 30 min) | Morin et al. (2009) |
| Sleep onset latency (SOL) | 30.00 | Above | minutes | Clinically elevated SOL (> 30 min) | Normal SOL (≤ 30 min) | Morin et al. (2009) |
| PSQI Global Score | 5.00 | Above | score (0-21) | Poor sleep quality (PSQI > 5) | Good sleep quality (PSQI ≤ 5) | Buysse et al. (1989) |
| ELISA CRP Levels | 1.10 | Above | log(mg/L) | Elevated CRP (> 3.0 mg/L; high cardiovascular risk) | Normal/low CRP (≤ 3.0 mg/L) | Pearson et al. (2003); Ridker et al. (2003) |
| ELISA IL-6 Levels | 1.14 | Above | log(pg/mL) | Elevated IL-6 (> 3.13 pg/mL; above MIDUS upper quartile) | Normal IL-6 (≤ 3.13 pg/mL) | Morozink et al. (2010) |
| MSD TNF-α Levels | 1.39 | Above | log(pg/mL) | Elevated TNF-alpha (> 4.0 pg/mL) | Normal TNF-alpha (≤ 4.0 pg/mL) | Danesh et al. (2014) |
| ELISA E-Selectin Levels | 3.94 | Above | log(ng/mL) | Elevated sE-Selectin (> 51.5 ng/mL; above MIDUS upper quartile) | Normal sE-Selectin (≤ 51.5 ng/mL) | Friedman et al. (2009) |
| ELISA Fibrinogen levels | 5.86 | Above | log(mg/dL) | Elevated Fibrinogen (> 350 mg/dL; elevated CVD risk) | Normal Fibrinogen (≤ 350 mg/dL) | Danesh et al. (2005) |
| ELISA ICAM-1 Levels | 5.52 | Above | log(ng/mL) | Elevated sICAM-1 (> 250 ng/mL) | Normal sICAM-1 (≤ 250 ng/mL) | Ridker et al. (1998) |
| MSD IL-6 Levels | 1.14 | Above | log(pg/mL) | Elevated IL-6/MSD (> 3.13 pg/mL) | Normal IL-6/MSD (≤ 3.13 pg/mL) | Morozink et al. (2010); platform-adjusted |
| MSD IL-8 Levels | 2.71 | Above | log(pg/mL) | Elevated IL-8 (> 15.0 pg/mL) | Normal IL-8 (≤ 15.0 pg/mL) | Yamamoto et al. (1997) |
| MSD IL-10 Levels | 2.21 | Above | log(pg/mL) | Elevated IL-10 (> 9.1 pg/mL; above population upper quartile) | Normal IL-10 (≤ 9.1 pg/mL) | Smith et al. (2018) |

*Note*. CRP = C-reactive protein; CVD = cardiovascular disease; ELISA = enzyme-linked immunosorbent assay; ICAM-1 = Intercellular Adhesion Molecule-1; IL = interleukin; MIDUS = Midlife in the United States; MSD = Meso Scale Diagnostics; PSQI = Pittsburgh Sleep Quality Index; SE = sleep efficiency; SOL = sleep onset latency; TNF-α = tumor necrosis factor-alpha; WASO = wake after sleep onset. Thresholds are expressed in log-transformed units consistent with the analytic approach used in this study. Direction indicates whether exceeding (Above) or falling below (Below) the threshold defines the clinically meaningful category. All thresholds are derived from published normative or clinical reference standards and are applied descriptively to contextualize latent profile differences; they were not used as classification criteria in the latent profile analysis.

## Table S11

*Descriptive Statistics for All 43 Study Variables in the Full Analytical Sample Prior to Latent Profile Classifications (n = 306)*

| **Variable** | ***M*** | ***(SD)*** | **Minimum** | **Maximum** | **Skewness** | **Kurtosis** |
| --- | --- | --- | --- | --- | --- | --- |
| Total Activity Count (TAC) | 7592.74 | (4204.04) | 1781.43 | 28696.57 | 1.44 | 2.99 |
| Activity Count Mean (ACM) | 17.65 | (9.87) | 4.63 | 74.10 | 1.76 | 4.73 |
| Maximum Activity Count (MAC) | 510.80 | (163.07) | 185.43 | 990.57 | 0.59 | -0.04 |
| Onset Latency (OL) | 25.07 | (22.13) | 0.21 | 128.57 | 1.88 | 3.74 |
| Snooze Time (SNT) | 13.44 | (14.55) | 0.50 | 79.00 | 2.22 | 5.52 |
| Sleep Efficiency (EFF) | 82.20 | (8.60) | 44.27 | 93.61 | -1.61 | 3.37 |
| Wake After Sleep Onset (WASO) | 44.24 | (19.75) | 11.50 | 139.86 | 1.13 | 2.29 |
| Wake Time (WT) | 44.27 | (19.80) | 9.86 | 139.86 | 1.13 | 2.28 |
| Percent Wake (PW) | 10.37 | (4.73) | 2.90 | 31.08 | 1.49 | 3.08 |
| Wake Bouts (WB) | 31.80 | (10.58) | 10.14 | 67.43 | 0.57 | 0.13 |
| Average Wake Bout (AWB) | 1.40 | (0.47) | 0.70 | 4.00 | 1.74 | 5.10 |
| Sleep Time (SLT) | 381.61 | (62.56) | 153.36 | 598.00 | -0.35 | 1.12 |
| Percent Sleep (PSL) | 89.62 | (4.74) | 68.92 | 97.21 | -1.48 | 3.04 |
| Sleep Bouts (SLB) | 32.12 | (10.59) | 10.14 | 66.71 | 0.56 | 0.06 |
| Average Sleep Bout (ASB) | 14.34 | (7.05) | 4.26 | 81.44 | 3.96 | 28.80 |
| MDD Severity | 0.62 | (1.69) | 0.00 | 9.00 | 2.64 | 5.89 |
| GAD Severity | 12.75 | (5.99) | 10.00 | 38.00 | 2.20 | 4.00 |
| Panic Disorder Severity | 0.38 | (1.12) | 0.00 | 6.00 | 3.30 | 10.81 |
| MSD IL-6 Levels | 0.69 | (0.38) | 0.17 | 3.93 | 3.25 | 19.50 |
| MSD IL-8 Levels | 2.73 | (0.36) | 1.83 | 4.91 | 1.12 | 5.04 |
| MSD IL-10 Levels | 0.25 | (0.18) | 0.08 | 1.59 | 4.09 | 21.72 |
| MSD TNF-α Levels | 1.16 | (0.22) | 0.67 | 2.29 | 0.86 | 2.19 |
| ELISA IL-6 Levels | 1.20 | (0.48) | 0.15 | 2.88 | 0.73 | 0.82 |
| ELISA Fibrinogen levels | 5.79 | (0.25) | 5.06 | 6.75 | 0.01 | 0.97 |
| ELISA CRP Levels | 1.05 | (0.71) | 0.10 | 4.14 | 1.36 | 2.89 |
| ELISA E-Selectin Levels | 3.70 | (0.50) | 0.92 | 5.09 | -0.57 | 2.60 |
| ELISA ICAM-1 Levels | 5.55 | (0.32) | 4.32 | 6.66 | -0.01 | 1.07 |
| Menopausal Drug Use Counts | 0.69 | (0.87) | 0.00 | 6.00 | 1.68 | 4.80 |
| Menstrual Symptom Counts | 1.26 | (0.71) | 0.00 | 3.00 | 0.07 | -0.31 |
| Aging Worries Count | 5.37 | (1.69) | 3.00 | 12.00 | 0.81 | 0.50 |
| PSQI 1 (Subjective Sleep Quality) | 0.97 | (0.66) | 0.00 | 3.00 | 0.65 | 1.29 |
| PSQI 2 (Sleep Latency) | 0.92 | (0.90) | 0.00 | 3.00 | 0.75 | -0.25 |
| PSQI 3 (Sleep Duration) | 0.80 | (0.79) | 0.00 | 3.00 | 0.88 | 0.51 |
| PSQI 4 (Habitual Sleep Efficiency) | 0.56 | (0.99) | 0.00 | 3.00 | 1.61 | 1.19 |
| PSQI 5 (Sleep Disturbances) | 1.26 | (0.54) | 0.00 | 3.00 | 0.76 | 0.68 |
| PSQI 6 (Use of Sleeping Medication) | 0.50 | (1.00) | 0.00 | 3.00 | 1.81 | 1.66 |
| PSQI 7 (Daytime Dysfunction) | 0.83 | (0.66) | 0.00 | 3.00 | 0.54 | 0.62 |
| Psychiatric diagnoses (count) | 0.17 | (0.46) | 0.00 | 2.00 | 2.76 | 6.87 |
| SUD severity | 78.42 | (14.71) | 28.00 | 89.00 | -2.89 | 6.40 |
| Chronic conditions (past 12 months) | 2.08 | (1.99) | 0.00 | 10.00 | 1.20 | 1.48 |
| Medications (past 30 days) | 5.21 | (18.84) | 0.00 | 98.00 | 4.68 | 20.11 |
| Supplements (past 30 days) | 1.82 | (2.00) | 0.00 | 15.00 | 1.96 | 6.73 |

*Note*. ACM = activity count mean; ASB = average sleep bout; AWB = average wake bout; CRP = C-reactive protein; ELISA = enzyme-linked immunosorbent assay; EFF = sleep efficiency; GAD = generalized anxiety disorder; ICAM-1 = Intercellular Adhesion Molecule-1; IL = interleukin; MAC = maximum activity count; MDD = major depressive disorder; MSD = Meso Scale Diagnostics; OL = onset latency; PSL = percent sleep; PSQI = Pittsburgh Sleep Quality Index; PW = percent wake; SLB = sleep bouts; SLT = sleep time; SNT = snooze time; SUD = substance use disorder; TAC = total activity count; TNF-α = tumor necrosis factor-alpha; WASO = wake after sleep onset; WB = wake bouts; WT = wake time. *M* = mean; *SD* = standard deviation. Descriptive statistics represent the full analytical sample before participants were assigned to latent profiles. This table is provided to characterize the overall sample and to enable assessment of variable distributions independent of profile membership. All variables are presented in their original, untransformed metric. Biomarker variables (MSD IL-6, MSD IL-8, MSD IL-10, MSD TNF-α, ELISA IL-6, ELISA Fibrinogen, ELISA CRP, ELISA E-Selectin, ELISA ICAM-1) were log-transformed prior to latent profile analysis and all regression models to address positive skewness; raw (untransformed) values are presented here for descriptive transparency. Variables with |skewness| > 1.00 or |kurtosis| > 3.00 are indicative of meaningful departure from normality.

## Table S12

*Sensitivity Analysis: Variable-Centered Regression Estimates for All 13 Latent Profile Analysis Indicators and Covariates Predicting Wave 2 MDD Severity (n = 306)*

| **Term** | **β** | **LCI** | **UCI** | ***p*** |
| --- | --- | --- | --- | --- |
| Intercept | 8.971 | -4.174 | 22.116 | .180 |
| Age (years) | -0.008 | -0.028 | 0.012 | .443 |
| Women vs. men | -0.245 | -0.708 | 0.218 | .299 |
| Multiracial | -1.386 | -4.105 | 1.332 | .316 |
| White | -2.555 | -3.982 | -1.129 | .001 |
| African American | -3.892 | -5.885 | -1.899 | < .001 |
| Native American or Aleutian Islander/Eskimo | -3.428 | -7.139 | 0.282 | .070 |
| Asian or Pacific Islander | -4.052 | -7.706 | -0.397 | .030 |
| Other | -3.035 | -5.794 | -0.276 | .031 |
| College education vs. High school | -0.019 | -0.501 | 0.462 | .937 |
| College education vs. No high school degree | -0.272 | -1.204 | 0.659 | .565 |
| College education vs. Some college | 0.170 | -0.317 | 0.657 | .493 |
| Baseline MDD severity | 0.085 | -0.120 | 0.289 | .416 |
| Chronic conditions (past 12 months) | 0.009 | -0.110 | 0.128 | .881 |
| Medications (past 30 days) | -0.006 | -0.017 | 0.004 | .230 |
| Supplements (past 30 days) | 0.134 | 0.030 | 0.238 | .012 |
| Psychiatric diagnoses (count) | 0.711 | -0.043 | 1.465 | .065 |
| SUD severity | -0.006 | -0.020 | 0.008 | .395 |
| Sleep efficiency (SE) | -0.033 | -0.082 | 0.016 | .182 |
| Wake after sleep onset (WASO) | -0.001 | -0.015 | 0.013 | .875 |
| Sleep onset latency (SOL) | -0.007 | -0.022 | 0.008 | .350 |
| PSQI Global Score | 0.075 | 0.011 | 0.139 | .023 |
| ELISA CRP Levels | 0.159 | -0.202 | 0.519 | .388 |
| ELISA IL-6 Levels | -0.192 | -1.059 | 0.675 | .664 |
| MSD TNF-α Levels | 0.043 | -1.320 | 1.407 | .950 |
| ELISA E-Selectin Levels | -0.327 | -1.684 | 1.029 | .635 |
| ELISA Fibrinogen levels | 0.421 | -4.920 | 5.762 | .877 |
| ELISA ICAM-1 Levels | -2.202 | -5.999 | 1.595 | .255 |
| MSD IL-6 Levels | 0.252 | -0.606 | 1.109 | .564 |
| MSD IL-8 Levels | 0.699 | -1.010 | 2.409 | .421 |
| MSD IL-10 Levels | -0.197 | -0.626 | 0.233 | .368 |

*Note*. CRP = C-reactive protein; ELISA = enzyme-linked immunosorbent assay; ICAM-1 = Intercellular Adhesion Molecule-1; IL = interleukin; LCI = lower bound of the 95% confidence interval; LPA = latent profile analysis; MDD = major depressive disorder; MSD = Meso Scale Diagnostics; PSQI = Pittsburgh Sleep Quality Index; SE = sleep efficiency; SOL = sleep onset latency; SUD = substance use disorder; TNF-α = tumor necrosis factor-alpha; UCI = upper bound of the 95% confidence interval; WASO = wake after sleep onset. β = unstandardized regression coefficient; *p* = probability value. This table presents the fully adjusted variable-centered regression model (Model 6) in which all 13 LPA indicator variables–three actigraphy sleep measures (SE, WASO, SOL), PSQI Global Score, and nine log-transformed biomarkers–were entered simultaneously alongside all demographic and clinical covariates. This model serves as a sensitivity benchmark against the primary LPA profile membership model (Model 5). Race/ethnicity contrasts are dummy-coded with a non-White, non-specified racial minority group as the implicit reference category for the omnibus comparison; individual contrasts reflect differences relative to the most prevalent reference group in context of the full model. Education contrasts use college education and above as the reference category. Statistically significant parameter estimates (*p* < .05) are indicated in bold. All biomarker variables are log-transformed. Confidence intervals are 95% and are not adjusted for multiplicity given the sensitivity and exploratory purpose of this model.

## Table S13

*Multiple Regression Estimates for Latent Profile Membership Predicting Wave 2 MDD Severity Nine Years Later, With Demographic Covariates and Baseline MDD Severity (Models 1–5) (n = 306)*

| **Model 1 (Unadjusted profile effect)** | ***b*** | ***(SE)*** | ***t*** | ***p*** | **Cohen’s *d*** |
| --- | --- | --- | --- | --- | --- |
| Intercept | 0.391 | (0.120) | 3.244 | .001 | 0.372 |
| Profile 2 vs. Profile 1 | 0.839 | (0.215) | 3.903 | < .001 | 0.448 |
| **Model 2 (+ Demographics)** | ***b*** | ***(SE)*** | ***t*** | ***p*** | **Cohen’s *d*** |
| Intercept | 3.538 | (0.884) | 4.000 | < .001 | 0.467 |
| Profile 2 vs. Profile 1 | 0.744 | (0.217) | 3.434 | .001 | 0.401 |
| Age (years) | -0.006 | (0.008) | -0.704 | .482 | -0.082 |
| Women vs. women | 0.141 | (0.203) | 0.691 | .490 | 0.081 |
| Multiracial | -1.943 | (1.413) | -1.376 | .170 | -0.161 |
| White | -2.934 | (0.720) | -4.076 | < .001 | -0.476 |
| African American | -3.467 | (1.001) | -3.465 | .001 | -0.405 |
| Native American or Aleutian Islander/Eskimo | -3.444 | (1.877) | -1.835 | .068 | -0.214 |
| Asian or Pacific Islander | -3.341 | (1.872) | -1.785 | .075 | -0.209 |
| Other | -3.180 | (1.417) | -2.244 | .026 | -0.262 |
| College education vs. High school | -0.064 | (0.249) | -0.259 | .796 | -0.030 |
| College education vs. No high school degree | -0.016 | (0.473) | -0.035 | .972 | -0.004 |
| College education vs. Some college | 0.180 | (0.248) | 0.728 | .467 | 0.085 |
| **Model 3 (+ Demographics + baseline depression)** | ***b*** | ***(SE)*** | ***t*** | ***p*** | **Cohen’s *d*** |
| Intercept | 3.010 | (0.866) | 3.474 | .001 | 0.407 |
| Profile 2 vs. Profile 1 | 0.458 | (0.220) | 2.081 | .038 | 0.244 |
| Age (years) | -0.001 | (0.008) | -0.137 | .891 | -0.016 |
| Women vs. women | 0.019 | (0.199) | 0.094 | .925 | 0.011 |
| Multiracial | -1.587 | (1.373) | -1.156 | .249 | -0.135 |
| White | -2.708 | (0.700) | -3.868 | < .001 | -0.453 |
| African American | -3.569 | (0.971) | -3.676 | < .001 | -0.430 |
| Native American or Aleutian Islander/Eskimo | -3.215 | (1.822) | -1.765 | .079 | -0.207 |
| Asian or Pacific Islander | -4.312 | (1.829) | -2.358 | .019 | -0.276 |
| Other | -2.913 | (1.376) | -2.117 | .035 | -0.248 |
| College education vs. High school | -0.037 | (0.241) | -0.153 | .879 | -0.018 |
| College education vs. No high school degree | 0.013 | (0.459) | 0.029 | .977 | 0.003 |
| College education vs. Some college | 0.266 | (0.241) | 1.103 | .271 | 0.129 |
| Baseline MDD severity | 0.270 | (0.061) | 4.399 | < .001 | 0.515 |
| **Model 4 (+ Health covariates)** | ***b*** | ***(SE)*** | ***t*** | ***p*** | **Cohen’s *d*** |
| Intercept | 3.085 | (0.861) | 3.582 | < .001 | 0.421 |
| Profile 2 vs. Profile 1 | 0.415 | (0.225) | 1.843 | .066 | 0.217 |
| Age (years) | -0.006 | (0.008) | -0.749 | .454 | -0.088 |
| Women vs. women | -0.150 | (0.207) | -0.724 | .470 | -0.085 |
| Multiracial | -1.658 | (1.362) | -1.217 | .224 | -0.143 |
| White | -2.661 | (0.695) | -3.828 | < .001 | -0.450 |
| African American | -3.631 | (0.968) | -3.751 | < .001 | -0.441 |
| Native American or Aleutian Islander/Eskimo | -3.638 | (1.835) | -1.983 | .048 | -0.233 |
| Asian or Pacific Islander | -4.154 | (1.827) | -2.274 | .024 | -0.268 |
| Other | -3.154 | (1.377) | -2.290 | .023 | -0.269 |
| College education vs. High school | 0.028 | (0.241) | 0.117 | .907 | 0.014 |
| College education vs. No high school degree | -0.047 | (0.459) | -0.103 | .918 | -0.012 |
| College education vs. Some college | 0.250 | (0.241) | 1.039 | .300 | 0.122 |
| Baseline MDD severity | 0.249 | (0.063) | 3.929 | < .001 | 0.462 |
| Chronic conditions (past 12 months) | 0.058 | (0.056) | 1.048 | .296 | 0.123 |
| Medications (past 30 days) | -0.006 | (0.005) | -1.155 | .249 | -0.136 |
| Supplements (past 30 days) | 0.112 | (0.051) | 2.177 | .030 | 0.256 |
| **Model 5 (Full Model + Clinical Covariates)** | ***b*** | ***(SE)*** | ***t*** | ***p*** | **Cohen’s *d*** |
| Intercept | 3.512 | (0.996) | 3.527 | < .001 | 0.416 |
| Profile 2 vs. Profile 1 | 0.396 | (0.225) | 1.765 | .079 | 0.208 |
| Age (years) | -0.005 | (0.008) | -0.581 | .562 | -0.069 |
| Women vs. women | -0.176 | (0.206) | -0.855 | .393 | -0.101 |
| Multiracial | -1.567 | (1.361) | -1.152 | .250 | -0.136 |
| White | -2.441 | (0.699) | -3.494 | .001 | -0.412 |
| African American | -3.570 | (0.966) | -3.696 | < .001 | -0.436 |
| Native American or Aleutian Islander/Eskimo | -3.434 | (1.828) | -1.879 | .061 | -0.222 |
| Asian or Pacific Islander | -3.854 | (1.822) | -2.115 | .035 | -0.250 |
| Other | -2.964 | (1.373) | -2.159 | .032 | -0.255 |
| College education vs. High school | 0.027 | (0.240) | 0.114 | .909 | 0.013 |
| College education vs. No high school degree | -0.193 | (0.462) | -0.419 | .676 | -0.049 |
| College education vs. Some college | 0.217 | (0.240) | 0.902 | .368 | 0.106 |
| Baseline MDD severity | 0.090 | (0.102) | 0.884 | .377 | 0.104 |
| Chronic conditions (past 12 months) | 0.038 | (0.057) | 0.666 | .506 | 0.079 |
| Medications (past 30 days) | -0.006 | (0.005) | -1.125 | .261 | -0.133 |
| Supplements (past 30 days) | 0.117 | (0.051) | 2.291 | .023 | 0.270 |
| Psychiatric diagnoses (count) | 0.708 | (0.370) | 1.914 | .057 | 0.226 |
| SUD severity | -0.009 | (0.007) | -1.242 | .215 | -0.147 |

*Note*. **p* < .05, ****p* < .001. AIC = Akaike Information Criterion; BIC = Bayesian Information Criterion; MDD = major depressive disorder. β = unstandardized regression coefficient; *F* = *F*-statistic; *N* = total sample size; *p* = probability value; *R*² = coefficient of determination. Unstandardized regression coefficients are presented with standard errors in parentheses. Model 1 enters latent profile membership (Profile 2 vs. Profile 1) as the sole predictor. Model 2 adds age (years) and sex (women vs. men) as demographic covariates. Model 3 additionally adjusts for baseline Wave 1 MDD severity to examine whether the profile effect persists after accounting for pre-existing depressive symptom load. A dash (–) indicates the predictor was not included in that model. The attenuation of the profile coefficient from Model 1 (β = 0.839) to Model 5 (β = 0.396) while retaining a meaningful effect size (Cohen’s *d* = 0.208) indicates that latent profile membership predicts nine-year MDD severity over and above baseline depression and demographic factors. Race/ethnicity was excluded from this model to maintain parsimony; full demographic covariate estimates including race/ethnicity and education are reported separately. p-values are presented without adjustment for multiplicity.

References

Buysse, D. J., Cheng, Y., Germain, A., Moul, D. E., Franzen, P. L., Fletcher, M., & Monk, T. H. (2010). Night-to-night sleep variability in older adults with and without chronic insomnia. *Sleep Medicine*, *11*(1), 56-64. <https://doi.org/10.1016/j.sleep.2009.02.010>

Buysse, D. J., Reynolds, C. F., 3rd, Monk, T. H., Berman, S. R., & Kupfer, D. J. (1989). The Pittsburgh Sleep Quality Index: a new instrument for psychiatric practice and research. *Psychiatry Research*, *28*(2), 193-213. <https://doi.org/10.1016/0165-1781(89)90047-4>

Danesh, A., Inglis, H. C., Jackman, R. P., Wu, S., Deng, X., Muench, M. O., . . . Norris, P. J. (2014). Exosomes from red blood cell units bind to monocytes and induce proinflammatory cytokines, boosting T-cell responses in vitro. *Blood*, *123*(5), 687-696. <https://doi.org/10.1182/blood-2013-10-530469>

Danesh, J., Lewington, S., Thompson, S. G., Lowe, G. D., Collins, R., Kostis, J. B., . . . Wood, A. (2005). Plasma fibrinogen level and the risk of major cardiovascular diseases and nonvascular mortality: an individual participant meta-analysis. *JAMA*, *294*(14), 1799-1809. <https://doi.org/10.1001/jama.294.14.1799>

Friedman, E. M., Williams, D. R., Singer, B. H., & Ryff, C. D. (2009). Chronic discrimination predicts higher circulating levels of E-selectin in a national sample: the MIDUS study. *Brain, Behavior, and Immunity*, *23*(5), 684-692. <https://doi.org/10.1016/j.bbi.2009.01.002>

Harvey, A. G. (2008). Insomnia, psychiatric disorders, and the transdiagnostic perspective. *Current Directions in Psychological Science*, *17*(5), 299-303. <https://doi.org/10.1111/j.1467-8721.2008.00594.x>

Hubert, L. J., & Arabie, P. (1985). Comparing partitions. *Journal of Classification*, *2*(2-3), 193-218. <https://doi.org/10.1007/BF01908075>

Love, G. D., Seeman, T. E., Weinstein, M., & Ryff, C. D. (2010). Bioindicators in the MIDUS national study: Protocol, measures, sample, and comparative context. *Journal of Aging and Health*, *22*(8), 1059-1080. <https://doi.org/10.1177/0898264310374355>

Mohd Razali, N., & Yap, B. (2011). Power comparisons of Shapiro-Wilk, Kolmogorov-Smirnov, Lilliefors and Anderson-Darling tests. *Journal of Statistical Modeling and Analytics*, *2*.

Morin, C. M., Vallières, A., Guay, B., Ivers, H., Savard, J., Mérette, C., . . . Baillargeon, L. (2009). Cognitive behavioral therapy, singly and combined with medication, for persistent insomnia: A randomized controlled trial. *JAMA*, *301*(19), 2005-2015. <https://doi.org/10.1001/jama.2009.682>

Morozink, J. A., Friedman, E. M., Coe, C. L., & Ryff, C. D. (2010). Socioeconomic and psychosocial predictors of interleukin-6 in the MIDUS national sample. *Health Psychology*, *29*(6), 626-635. <https://doi.org/10.1037/a0021360>

Muthén, L. K., & Muthén, B. O. (2002). How to use a Monte Carlo study to decide on sample size and determine power. *Structural Equation Modeling*, *9*(4), 599-620. <https://doi.org/10.1207/s15328007sem0904_8>

Nylund-Gibson, K., & Choi, A. Y. (2018). Ten frequently asked questions about latent class analysis. *Translational Issues in Psychological Science*, *4*(4), 440-461. <https://doi.org/10.1037/tps0000176>

Ohayon, M., Wickwire, E. M., Hirshkowitz, M., Albert, S. M., Avidan, A., Daly, F. J., . . . Vitiello, M. V. (2017). National Sleep Foundation's sleep quality recommendations: first report. *Sleep Health*, *3*(1), 6-19. <https://doi.org/10.1016/j.sleh.2016.11.006>

Pearson, T. A., Mensah, G. A., Alexander, R. W., Anderson, J. L., Cannon, R. O., 3rd, Criqui, M., . . . Vinicor, F. (2003). Markers of inflammation and cardiovascular disease: application to clinical and public health practice: A statement for healthcare professionals from the Centers for Disease Control and Prevention and the American Heart Association. *Circulation*, *107*(3), 499-511. <https://doi.org/10.1161/01.cir.0000052939.59093.45>

Ridker, P. M., Buring, J. E., Cook, N. R., & Rifai, N. (2003). C-reactive protein, the metabolic syndrome, and risk of incident cardiovascular events: an 8-year follow-up of 14 719 initially healthy American women. *Circulation*, *107*(3), 391-397. <https://doi.org/10.1161/01.cir.0000055014.62083.05>

Ridker, P. M., Hennekens, C. H., Roitman-Johnson, B., Stampfer, M. J., & Allen, J. (1998). Plasma concentration of soluble intercellular adhesion molecule 1 and risks of future myocardial infarction in apparently healthy men. *Lancet*, *351*(9096), 88-92. <https://doi.org/10.1016/s0140-6736(97)09032-6>

Smith, L. K., Boukhaled, G. M., Condotta, S. A., Mazouz, S., Guthmiller, J. J., Vijay, R., . . . Richer, M. J. (2018). Interleukin-10 directly inhibits CD8(+) T cell function by enhancing N-glycan branching to decrease antigen sensitivity. *Immunity*, *48*(2), 299-312 e295. <https://doi.org/10.1016/j.immuni.2018.01.006>

Steinley, D. (2004). Properties of the Hubert-Arable Adjusted Rand Index. *Psychological Methods*, *9*(3), 386-396. <https://doi.org/10.1037/1082-989X.9.3.386>

Yamamoto, C., Yoneda, T., Yoshikawa, M., Fu, A., Tokuyama, T., Tsukaguchi, K., & Narita, N. (1997). Airway inflammation in COPD assessed by sputum levels of interleukin-8. *Chest*, *112*(2), 505-510. <https://doi.org/10.1378/chest.112.2.505>
